# Cadmium toxicity in medicinal plants: An overview of the tolerance strategies, biotechnological and omics approaches to alleviate metal stress

**DOI:** 10.3389/fpls.2022.1047410

**Published:** 2023-01-17

**Authors:** Jameel M. Al-Khayri, Akshatha Banadka, R Rashmi, Praveen Nagella, Fatima M. Alessa, Mustafa I. Almaghasla

**Affiliations:** ^1^ Department of Agricultural Biotechnology, College of Agriculture and Food Sciences, King Faisal University, Al-Ahsa, Saudi Arabia; ^2^ Department of Life Sciences, CHRIST (Deemed to be University), Bangalore, Karnataka, India; ^3^ Department of Food Science and Nutrition, College of Agriculture and Food Sciences, King Faisal University, Al-Ahsa, Saudi Arabia; ^4^ Department of Arid Land Agriculture, College of Agriculture and Food Sciences, King Faisal University, Al-Ahsa, Saudi Arabia; ^5^ Plant Pests, and Diseases Unit, College of Agriculture and Food Sciences, King Faisal University, Al-Ahsa, Saudi Arabia

**Keywords:** cadmium, medicinal plants, transporters, reactive oxygen species, plant secondary metabolites, CRISPR- Cas 9

## Abstract

Medicinal plants, an important source of herbal medicine, are gaining more demand with the growing human needs in recent times. However, these medicinal plants have been recognized as one of the possible sources of heavy metal toxicity in humans as these medicinal plants are exposed to cadmium-rich soil and water because of extensive industrial and agricultural operations. Cadmium (Cd) is an extremely hazardous metal that has a deleterious impact on plant development and productivity. These plants uptake Cd by symplastic, apoplastic, or *via* specialized transporters such as HMA, MTPs, NRAMP, ZIP, and ZRT-IRT-like proteins. Cd exerts its effect by producing reactive oxygen species (ROS) and interfere with a range of metabolic and physiological pathways. Studies have shown that it has detrimental effects on various plant growth stages like germination, vegetative and reproductive stages by analyzing the anatomical, morphological and biochemical changes (changes in photosynthetic machinery and membrane permeability). Also, plants respond to Cd toxicity by using various enzymatic and non-enzymatic antioxidant systems. Furthermore, the ROS generated due to the heavy metal stress alters the genes that are actively involved in signal transduction. Thus, the biosynthetic pathway of the important secondary metabolite is altered thereby affecting the synthesis of secondary metabolites either by enhancing or suppressing the metabolite production. The present review discusses the abundance of Cd and its incorporation, accumulation and translocation by plants, phytotoxic implications, and morphological, physiological, biochemical and molecular responses of medicinal plants to Cd toxicity. It explains the Cd detoxification mechanisms exhibited by the medicinal plants and further discusses the omics and biotechnological strategies such as genetic engineering and gene editing CRISPR- Cas 9 approach to ameliorate the Cd stress.

## 1 Introduction

Extensive urbanization and expeditious industrialization have primarily contributed to environmental pollution. Environmental pollutants such as inorganic pollutants (including heavy metals), gaseous pollutants, organic and organometallic compounds, radioactive isotopes, and toxicity of some nanoparticles have been polluting the environment (Yadav, 2010). In spite of a worldwide focus on overcoming pollution, it has become a major challenge to be faced due to its dreadful long-term consequences. Environmental pollution has become one of the prominent causes of distress and mortality worldwide. Of all the pollutants, the inorganic heavy metal pollutants have gained special attention due to their omnipresent occurrence and their toxic effects (Benavides et al., 2005).

Heavy metals are high atomic weight elements with a density five times greater than that of water (Tchounwou et al., 2012). There are essential and non-essential heavy metals. The essential heavy metals are required in trace amounts. They are essential for plant growth, animals, and the human body and take part in electron transport, redox reactions, and nucleic acid metabolism (Narender, 2005). However, when these metals accumulate beyond the tolerable limits, they pose a serious threat disturbing the normal functioning of biological organisms. Heavy metals such as Iron (Fe), Molybdenum (Mo), and Manganese (Mn) serve as micronutrients. Heavy metals such as Chromium (Cr), Cobalt (Co), Copper (Cu), Nickel (Ni), Vanadium (Vn), and Zinc (Zn) are needed in trace quantities. However, they can be toxic when they are found in higher concentrations. Some non-essential heavy metals like Antimony (Sb), Arsenic (As), Cadmium (Cd), Lead (Pb), Mercury (Hg), and Silver (Ag) have no biological functions and seem to be toxic to organisms (Benavides et al., 2005). The heavy metal pollutants get into the water and soil through anthropogenic sources like agricultural fungicide and pesticide runoff, domestic garbage dumps, industrial effluents, mining operations, sewage sludges, and urban composts (Srivastava et al., 2017).

The plants grown in such heavy metal contaminated sites or irrigated with heavy metal contaminated water take up the metals. These heavy metal contaminated plants when consumed by animals and humans enter and disturb the food chain (Gall et al., 2015). Thus, heavy metals uptake by plants increases the possibility of these toxic elements entering the food chain. In recent times, heavy metal toxicity studies in medicinal plants have been a topic of considerable interest. Cadmium, one of the heavy metals with extreme toxicity has negatively impacted the plant development and productivity (Patel, 2006a).

Medicinal plant use in traditional medicine and ethnomedicine is a long-standing tradition. Medicinal plants are rich in therapeutic bioactive molecules that can be used to combat a wide variety of diseases. These bioactive molecules are synthesized *via* different metabolic pathways. They possess anticancer, antidiabetic, diuretic, antihypertensive, anti-inflammatory, antimicrobial, hypolipidemic, and many more properties. Medicinal plants and their products have been used in the treatment of lifestyle disorders such as cardiovascular diseases, diabetes, hypertension, inflammatory diseases, mental disorders and skin diseases (Oladeji, 2016; So et al., 2018). Plant-derived herbal medicines are preferred over western medicine and their usage has substantially increased with time. About 60% of the world population with 80% African, 80% Arabians, 48% Australian, 39% Belgium, 30-50% Chinese, 70% Canadian, 76% French, 80% Germans, 70% Indians and 42% USA people rely on herbal medicines (El-Dahiyat et al., 2020; Saggar et al., 2022; Bahl, 2022). It is expected that the global trade of medicinal plants would reach 5 trillion USD by 2050 (Zahra et al., 2020). Although medicinal plant-derived herbal products are gaining more popularity, the safety of use of such products has become a major concern. The herbal products derived from these medicinal plants have shown heavy metal toxicity due to contamination during cultivation, cross-contamination, or deliberate introduction of heavy metals (Street, 2012). When assessed for the heavy metal contamination in *Menthae piperitae* and *Anthodium chamomillae*, nearly 14-16% of cadmium content which exceeded the acceptable limits of World Health Organization (WHO) standards (10 mg/kg) (Mirosławski and Paukszto, 2018).

Plants grown in heavy metal contaminated sites have adopted different mechanisms to fight stress. They can either be sensitive to heavy metal contamination showing injury or death as a response to stress or they can exhibit coping mechanisms to stress by tolerance or avoidance. Avoiders are those plants that prevent the entry of metal ions into the plant whereas the tolerant plants detoxify the metal ions that have entered the plant system. Based on these strategies they are broadly classified as hyperaccumulators, metal excluders, and indicators (Mehes-Smith et al., 2013). Plants that are sensitive to metals show physiological, biochemical, and genetic changes causing delayed seed germination, stunted growth, chlorosis, limped leaves, less branching, less fruiting, and many more abnormalities (Haque et al., 2021). The tolerant plants release cellular and root exudates as the first line of defense against heavy metal uptake. As a second line of defense, they chelate, sequester, and detoxify the heavy metals. The plants under heavy metal stress produce antioxidants, stress-related hormones, and proteins (Ghori et al., 2019). Heavy metal stress can induce changes in secondary metabolite (SM) production (Nasim and Dhir, 2010; Asgari-Lajayer et al., 2017).

Comprehensive documentation exists on the effects of different heavy metals on plant physiology and their biochemistry in crop plants. But not much attention has been given to the effects of heavy metals on active SMs of medicinal plants. It is, therefore, necessary to evaluate the effect of heavy metals in medicinal plants. Of the various known heavy metals, Cd is one of the most treacherous metals due to its high mobility and toxicity at lower concentrations (Benavides et al., 2005). Taking this into account, the present review discusses the effect of Cd on seed germination, plant growth, physiological characteristics, and biochemical aspects, with an emphasis on the biosynthesis of important SMs in medicinal plants. The review discusses the defense mechanisms and detoxification strategies exhibited by the plants to combat Cd stress. The omics approaches and various biotechnological approaches like genetic engineering approach, and CRISPR Cas 9 gene editing approach for enhancing the ability of plants to survive the Cd stress has been covered.

## 2 Sources of cadmium

Cadmium is a heavy metal with atomic number 48 designated as Cd. It is a bluish-white, malleable soft metal. It naturally occurs in the environment as a natural cadmium sulfide ore or is found in association with zinc. It is a nonessential heavy metal to biological organisms that are known to cause toxic effects in excretory, gastrointestinal, neurological, reproductive, respiratory, and skeletal systems and negatively affect plant growth. Because of its high toxicity and high solubility in water, Cd has been regarded as a significant pollutant. A soil is considered to be non-polluted, if the Cd levels are between 0.04 to 0.32 mM (Wagner, 1993). However, if the Cd levels cross 0.32 mM and go up to 1 mM the soil is considered to be moderately polluted. Drinking water with Cd level below 1 ppb is considered to be potable (Sanità di Toppi and Gabbrielli, 1999).

The main source of Cd pollution in the environment includes smelting and mining activities both of which can pollute the air with Cd. The Cd compounds can associate with air-borne particles and can be carried across long-distance which then gets deposited into the soil by rain. Incineration of municipal waste, industrial runoffs from metal, pigment-producing, and battery manufacturing industries, contamination with sewage sludges, seepage from waste sites, chemical fertilizers pollutes the soil and water with Cd (Kubier et al., 2019). The underground water is known to be contaminated by mining, the release of industrial effluents, or by seepage from hazardous sites. Once the Cd enters soil and water it can easily get into the food chain through plants which is a major concern (ATSDR, 2013).

## 3 Cadmium mobilization

**Figure 1:**
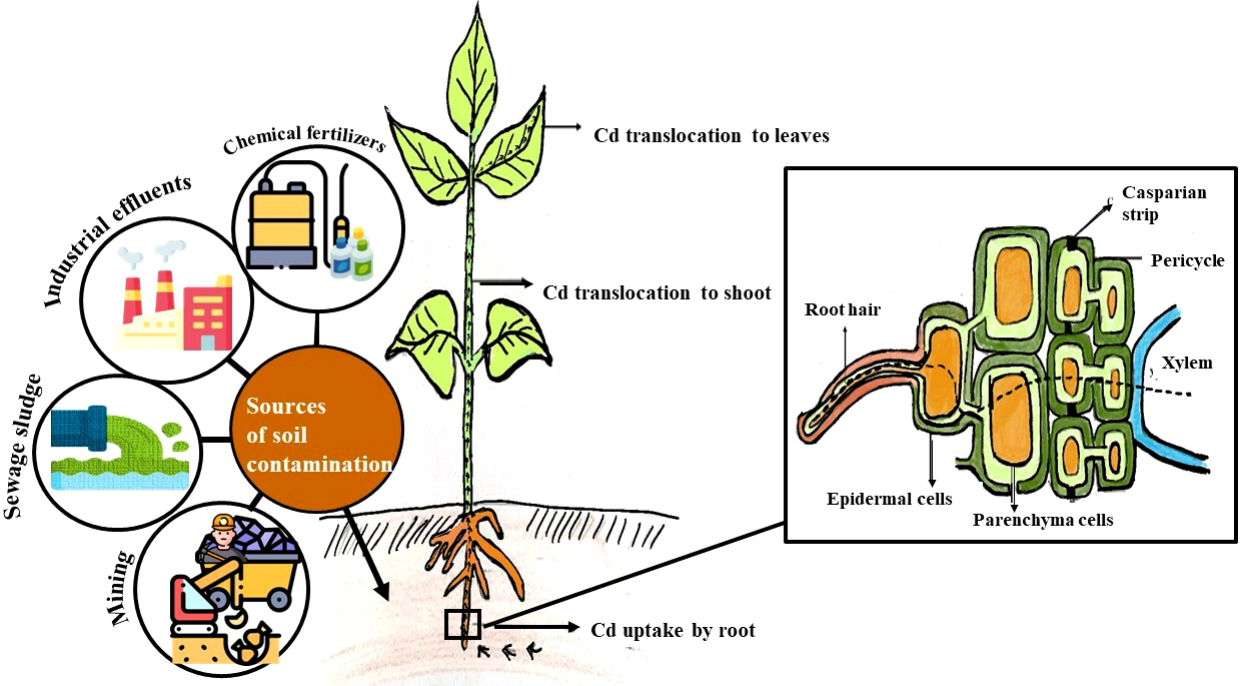
Source, uptake and translocation of cadmium from soil to root, shoot and leaves and mechanism underlying the mobilization of cadmium through root hairs to the xylem vessel.

The Cd uptake and transfer in plants depend on the ability of the plants to absorb the metals. Some plants resist the uptake of metal, while some facilitate the metal uptake. The Cd uptake is also affected by the metal concentration of soil, the physicochemical properties of soil such as temperature, pH, and redox potential as well as other components including the organic matter of the soil. The uptake and translocation of Cd by plants are represented in **Figure 1**. The Cd metals gain entry into the plant and are transported within the plant through the different membranes at various levels through non-selective cationic channels or through other metal transporters (Huang et al., 2020). The Cd uptake and translocation in plants take place through apoplastic and symplastic pathways (Song et al., 2017). Roots are the first part of the plant that comes in contact with heavy metals in soil. The Cd in the soil solution gets onto the root surface through root hairs which serve as an active zone of absorption and epidermal cells through ion exchange. Root secretes low molecular compounds such as mugineic acids which chelate Cd^+^ and facilitate its transport. It is then transported into the parenchyma cells across the root cortex through the Casparian strip in the endodermis (Song et al., 2017). Once inside the parenchymal cells, the Cd ion enters the conductive vessels of the xylem through the symplast. Thus, Cd enters into the xylem *via* apoplastic or symplastic pathways. The roots can retain the heavy metals or it can facilitate the metal movement into the shoot. The root cells retain Cd by insolubilization at the root surface and apoplast or avoid the release to the xylem by compartmentation in cells (Page and Feller, 2015). The mechanism underlying the mobilization of cadmium through root hairs to the xylem vessel is represented in **Figure 1**. The heavy metals in the roots are transported to transpiring shoot parts (leaves and stems) through the transpiration stream in the xylem. The chelated or free metal ions move upwards along the xylem sap. The heavy metal concentration in the transpiration stream of the root xylem depends upon the cell wall interaction of xylem vessels during transport. The heavy metals would either accumulate in leaves if there is no further redistribution. The Cd in the leaf cell cytosol is chelated by organic ligands, and it can move to adjacent cells, some of which get accumulated in the vacuole. The heavy metals get redistributed by a symplastic pathway to the other growing plant parts *via* the phloem. The Cd ions also move and accumulate in the reproductive organs, developing fruits and seeds. It might get redistributed to roots where Cd could be expelled (Page and Feller, 2015; Sterckeman and Thomine, 2020).

Diversified groups of metal transporters present in the plasma membrane facilitate the translocation of Cd through the symplastic and apoplastic pathways. The extracellular location and the biological function of these transporters are still unclear. There are four major Cd transporters which include heavy metal transporting ATPase transporter protein (HMA), metal tolerance or transporter proteins (MTPs), the NRAMP (natural resistance-associated macrophage protein), and the ZIP (zinc-regulated transporters, iron-regulated transporter-like proteins/ZRT-IRT-like Proteins) families. Heavy metal ATPase transport protein is a subgroup of the large P-type ATPase family that transports divalent Cd^2+^ ions between cytoplasm, cellular compartments, and xylem. They help in the efflux of heavy metals from the cytoplasm across the plasma membrane or into other organelles. Transporters like NRAMP6 (natural resistance-associated macrophage protein 6), IRT1 (iron-regulated transporter 1) in *Brassica napus* L. take part in Cd accumulation (Chen et al., 2018b). HMA transporters like AtHMA2, and OsHMA2 are HMAs that are located in the plasma membrane, and also take part in the translocation of Cd ions from root to shoots by loading Cd ions into the xylem. Another important HMA, AtHMA1, located in the inner chloroplast membrane, also helps in Cd transport (Fan et al., 2018).

Metal transporter proteins (MTPs) are a group of membrane-bound proteins that belong to the Cation Diffusion Facilitator (CDF) family. The MTPs located in the tonoplast act as antiport and mediate in the transport of divalent cations. Hence, they are also referred to as cation efflux transporters. They help in resisting or tolerating the Cd stress by the sequestration and efflux of Cd^2+^ ions. Nearly eleven MnMTPs are identified as Transporter proteins in *Morus notabilis* which helps in heavy metal transport (Fan et al., 2018). Natural resistance-associated macrophage proteins, abbreviated as NRAMP (Sasaki et al., 2012) are a group of proteins that transport a variety of metals. These proteins help in the translocation of metals from the root to shoot across the cell membrane and vacuolar membrane. *Arabidopsis thaliana* is known to transfer Cd metal and the AtNramp3 gene is known to enhance Cd resistance of root growth translocation of divalent cations across membranes (Thomine et al., 2000). Iron-regulated transporter-like proteins (ZIP) and Zinc-regulated transporters are principal metal transporters. The first identified proteins from their family of transporters were Zinc-regulated transporters and iron-regulated transporter-like proteins, hence the name ZIP. These proteins are also engaged in the displacement of divalent cations through plasma membranes. ZIP transporters like AtIRT1 in *A. thaliana*, NcZNT1 in *Noccaea caerulescens* (J. & C. Presl) F.K. Meyer, OsIRT1, OsIRT2, OsZIP6 in *Oryza sativa*, MtZIP6, OsNRAMP1, OsNRAMP5 in *Medicago truncatula*, and NRAMP5 in *Hordeum vulgare* all of which are localized on the plasma membrane mediates Cd^2+^ (Komal et al., 2015).

## 4 Cadmium toxicity

### 4.1 Impact of Cadmium on various growth stages

The study of heavy metal effects on different growth stages is important to understand the extent to which heavy metals limit plant growth and productivity in general (Patel, 2006a). It also helps to know the possible mechanisms the plants employ to survive the heavy metal stress at different plant growth stages. The ability of plants to uptake, transfer and accumulate heavy metals varies with different stages of plant growth. It is generally known that young seedlings have the ability to uptake metals at a higher rate when compared to mature plants (Souri et al., 2019). Under heavy metal stress, the plants might show delayed germination and exhibit poor vegetative growth at multiple levels with anatomical, morphological and biochemical changes. Plants at reproductive stages are known to be even more sensitive to metal stress (Ma et al., 2020).

#### 4.1.1 Germination

Seed germination is the initial stage and one of the most crucial stages in the plant life cycle. The seed, during its germination, is highly susceptible to the physiological conditions of the rhizosphere (Bewley, 1997). Despite the outermost seed coat covering serving as a protective barrier against the detrimental effects of heavy metals, the metal stress induces slow germination and suppresses response vigor in seeds. Heavy metals suppress seed germination and seedling development by inhibiting food storage and mobilization, morphological changes like reduction in radical and plumule formation, and modification in proteolytic activities. Thus, the consequences of heavy metals on seedling germination and growth must be widely studied (Seneviratne et al., 2019). The effect of different concentrations of Cd ranging from 0- 16 mg L^−1^ has been studied in *Ocimum basilicum* L. The germination percentage was reduced to 4% with the lowest germination at a Cd concentration of 16 mg L^−1^ (Fattahi et al., 2019). In the study conducted by Khatamipour et al. (2011), the effect of different concentrations of cadmium chloride varying from 0-600 mg L^−1^ was studied on the germination in *Silybum marianum*. The increase in Cd concentration showed a noticeable reduction in germination, the shoot and root length of seedlings, and proline content with the lowest. It has been reported to hamper food reserve mobilization due to the disturbance in sugars and amino acids causes mineral leakage leading to nutrient loss, over-accumulation of lipid peroxidation products, inhibition of alpha-amylase and invertases activity all of which resulting in delayed and reduced seed germination (Sethy and Ghosh, 2013). The effect of cadmium on germination in various plants is represented in **Table 1**.

**Table 1:** Effect of cadmium on germination, vegetative and reproductive growth in medicinal plants.

Plant name	Metal concentration	Effect on germination	Effect on vegetative growth	Effect on reproductive growth	Reference
*Adhatoda vasica* L.	0,100, 200, 300, 400, 500, 600 ppm	–	Increasing Cd conc. had inhibitory effect on elongation, fresh and dry weight of root and shoot RRG value, leaf number, fresh weight, and area	Number, dry weight, fresh weight of inflorescence, flower bud, fruit reduced	(Trivedi, 2003)
*Alternanthera tanella* Colla	0, 50, 100 or 150 μM	–	The shoots and roots reduced with increasing concentration	–	(Rodrigues et al., 2017)
*Amaranthus spinosus* L.	5-50 ppm for 60 days	–	Significant reduction in root and shoot length and fresh weight in dose dependent manner	–	(Huang Y et al., 2019)
*Andrographis paniculata* (Burm.f.) Nees	10,50,100,150and 200 ppm	–	Root and stem elongation, RRG values. leaf number, dry and fresh weight of root, stem, and leaf was gradually lowered and percent phytotoxicity values increased with increasing in metal concentration	Inflorescence branch number pollen tube growth and pollen germination, flower, flower bud and fruit number n fresh weight of inflorescence and flower bud decreased	(Patel, 2006a)
*Anethum graveolens* L.	0, 100 and 200 μM	–	Root length, leaf area, shoot and root dry weight decreased	–	(Aghaz et al., 2013)
*Bacopa monnieri* L.	5 μM, 10 μM, 50 μM, and 100 μM	–	Browning and stunting of roots with decreased biomass were observed with increasing Cd concentration	–	(Gupta et al., 2014)
*Bidens pilosa* L.	2.57ppm, 7.94 ppm, 17.33ppm, and 37.17ppm for 40 days	–	Root and shoot biomasses gradually decreased with increasing concentration	–	(Dai et al., 2021)
*Brassica juncea* L.	200 mg L^-1^ and 300 mg L^-1^	–	Plant height, root length and biomass reduced	–	(Ahmad et al., 2015)
*Cannabis sativa* L.	25 mg kg^-1^ Cd for 45 days	–	Shoot and root biomass decreased with increasing concentration	–	(Shi et al., 2012)
*Catharanthus roseus* var. *rosea* L.	(0, 10, 50, 100, 200, 500 and 1000 µM	0% germination at 1000 µM concentration	The root length was inhibited	–	(Pandey et al., 2007)
*Cajanus cajan* L.	1,5,10,20,50mg L^−1^	60% reduction in seed germination with a decrease in the fresh and dry weight reduction in growth, stunting of seedlings	Reduction in fresh and dry weight and stunting of seedlings	–	(Patnaik and Mohanty, 2013)
*Centella asiatica* L.	50-100 ppm for 30 days	–	The root length remained the same except at 100 ppm while the shoot length increased significantly with metal concentration	–	(Biswas et al., 2020)
*Cichorium pumilum* Jacq.	50, 100, 200, 400, 800, and 1600 µM	–	Hypocotyl and root length decreased with increasing Cd concentration	–	(Khateeb, 2013)
*Coriandrum sativum* L.	0, 25, 50, and 100 mg kg^-1^	Germination % (least at 50mg kg^−1^ Cd)	Root length, shoot length decreased with an elevation of Cd conc. with least at 100mg kg^-1^Cd	–	(Faizan et al., 2012)
*Cuminum cyminum* L.	0, 300, 450, 600, 750 and 1050 µM	30% and 23% inhibition in seed germination of Isfahan and Khorasan ecotypes respectively.	43.6% and 48.7% of root growth inhibition of Isfahan and Khorasan ecotypes respectively.	–	(Salarizadeh et al., 2016)
*Drimia elata* Jacq. ex Willd.	2, 5, 10 mg L^-1^	–	The shoot and bulb dry weight reduced significantly with higher concentrations	–	(Okem et al., 2015)
*Melissa officinalis* L.	0, 10, 20 and 40 μM	–	Fresh weight increased upto 20 μM	–	(Nourbakhsh Rezaei et al., 2019)
*Merwilla plumbea* (Lindl.) Speta	1.5 ppm	–	The fresh weight of leaves, bulbs and roots significantly reduced	–	(Lux et al., 2011a)
*Moringa oleifera* Lam.	1- 5 mM for 30 days	–	The root and shoot length significantly reduced	–	(Srivastava and Yadav, 2017)
*Ocimum basilicum* L.	5, 10, 15, 20, 25 ppm	–	The fresh and dry weight declined with increasing Cd concentration	–	(Youssef, 2021)
	0-16 mg L^−1^	4% reduction in germination at 16 mg L−1 Cd	–	–	(Fattahi et al., 2019)
*Ocimum canum* Sims.	50, 100, 150, 200, 250 mg kg^-1^	–	The root elongation and stem height inhibited	Flower number and its fresh weight and dry weight, inflorescence, fruit number. dry weight	(Patel, 2006b)
*Phyllanthus amarus* Schumach. and Thonn.	10-100 mg kg^-1^	–	The root and shoot growth of plant remained unaffected upto 50 ppm and further decreased with the increasing concentration.	–	(Dwivedi et al., 2013)
*Silybum marianum* L. Gaertn.	0, 100, 200, 400 and 600 mg L^-1^	14% seed germination at 600 mg L^-1^	–	–	(Khatamipour et al., 2011)
*Trigonellafoenum-graecum* L.	0.1, 0.5, 1 and 10 mM	33% decrease in germination and no radicle growth at 10 mM	–	–	(Zayneb et al., 2015)
*Trigonella foenum-graecum* L.	0, 5, 15, 30, 50 μg g ^-1^	–	Magnitude of increase of number of leaves, leaf area and number of branches per plant, along with shoot and root length was lowered	–	(Ahmad et al., 2005)
*Typha latifolia* L.	0.2–0.8 μg g ^-1^	–	Leaf, shoot and root elongation and the dry weight reduced	–	(Ye et al., 1997)
*Withania somnifera* L. Dunal.	50- 1,000 μM	–	FW and DW was almost same at lower and moderate concentrations and drastically decreased at higher concentration	–	(Mishra et al., 2014)

#### 4.1.2 Vegetative stage

The vegetative stage indicates a period of growth between germination and flowering stages of plant growth during which plants are involved in producing leaves, stems, and branches without flowers (Hangarter, 2000; Gilbert, 2000). Heavy metals like Cd have a wide range of harmful effects on the vegetative stage of plants causing chlorosis, inhibition of photosynthesis, low biomass accumulation, retardation of growth, altered osmoregulation, changes in nutrient assimilation, and senescence, which ultimately results in plant death. Plants of various sorts have diverse development tendencies and respond differently to heavy metal stress (Singh et al., 2015). The metal has been generally known to decrease plant height and biomass. In the heavy metal studies conducted on *Coriandrum sativum* L. it has been observed that the root and shoot length reduced drastically with increasing concentration. The study suggests that the Cd metal accumulated in the root slowed down the mitotic rate in meristematic cells leading to reduced root length. On the other hand, the shoot length is reduced due to a reduction in the meristematic cells. The other possible reason for the reduction in the root and shoot length is due to the action of cotyledonary enzymes that digest carbohydrates and protein in the radical and plumule tips (Faizan et al., 2012). The effect of Cd (5, 10, 50, and 100 *μ*M) on different stages of growth in *Bacopa monnieri* has been studied and it has been observed that the biomass reduced with increasing cadmium (Gupta et al., 2014). The various effects of Cd on plant growth are represented in **Table 1**.

#### 4.1.3 Reproductive stage

The reproductive stage of plant growth involves the development of flower buds and flowers. The flower, on fertilization, develops into fruit with seeds. The heavy metals induce delayed flowering and fruiting and decrease their yield. This, in turn, decreases flowering and fruiting indices (Shekari et al., 2019). In the study conducted on *Andrographis paniculata*, Cd suppressed the reproductive growth by decreasing the number in the inflorescence branch, flower, and flower buds, and also suppressed the fresh weight of the inflorescence and flower bud (Patel, 2006a). Cadmium affects the production and allocation of amino acids and sugars, absorption, assimilation, and distribution of nutrients in plants (Carvalho et al., 2021). The fruit number and the fruit biomass has reported to be reduced in *Adhatoda vasica* L. grown in Cd treated soil when compared to control. The reduction in fruit number could be attributed to the loss of important nutrients like K, Fe and Zn. Further the reduction in fresh weight of inflorescence resulted in poor seed development (Trivedi, 2003). The effect of Cd on reproduction in medicinal plants is presented in **Table 1**.

### 4.2 Anatomical changes

Plant morphology, physiology, and anatomy are likely to reveal information about their ability to adapt to different growth environments. Under Cd toxicity, plants show various anatomical changes, especially in root tissues. Since roots are often the first organs to be exposed to metal ions, they always try to avoid the Cd lodgment in shoots by limiting their entry either by symplastic or by apoplastic pathways. When plant roots are subjected to high Cd levels, they release phytochelatins to sequester Cd as Cd-chelates in the vacuole of root cells and thus prevent symplastic entry. Meanwhile, they hasten the maturation of endodermis by bearing suberin lamellae, Casparian bands, and lignification near to the root apex to avoid the apoplastic entry of cd (Lux et al., 2011b). The development of hypodermal Suberin-impregnated periderm with impermeable cell walls periderm in the immature sub-apical areas of *Merwilla* roots acts as a defense response of roots that may inhibit radial Cd ion absorption by roots (Lux et al., 2011a). The effect of Cd on plant anatomy is presented in **Table 2**.

**Table 2:** Anatomical changes in cadmium treated medicinal plants.

Plant	Metal concentration	Anatomical changes	Reference
*Alternanthera tenella* Colla.	50, 100, 150 ppm	Endodermal and ectodermal wall thickening in roots, damaged inner root cells, reduced epidermal thickness in both adaxial and abaxial surfaces.	(Rodrigues et al., 2017)
*Brassica juncea* L.	50ppm, 500 ppm,2.5 mM, and 10 mM	Precipitation along cell walls and an increase in the number of vacuoles in root cortical cells, black depositions along the walls of vascular bundles of stems	(Sridhar et al., 2005)
*Melissa officinalis* L.	10,20,30 ppm	Decreasing number and size of stomata and epidermal cells with increasing Cd concentration	(Kilic, 2017)
*Merwilla plumbea* (Lindl.) Speta	1,5 ppm	Hypodermal periderm formation near to root apex.	(Lux et al., 2011a)
*Salvia sclarea* L.	0-100ppm	Reduced epidermal cell size, spongy parenchyma and mesophylls with less intercellular space	(Dobrikova et al., 2021)
*Thlaspi caerulescens* J. & C. Presl	0.5 to 500 ppm	Damaged cells, irregular intracellular space in root cortex	(Wójcik et al., 2005)
*Trigonella foenum-graecum* L.	5,15,30,50 ppm	Reduced stomatal density, decreased proportion of pith and vasculature and increased pith and cortex ratio in stem, decreased density and dimensions of xylem vessels in both root and shoot	(Ahmad et al., 2005)

Since leaf is an important site for photosynthesis and leaf morphology and anatomy have vital roles in photosynthetic efficiency. Leaf characteristics like thickness and stomatal density impact metal tolerance and sensitivity directly (Thongchai et al., 2021). Anatomical changes in leaves can reflect biological activity in plants associated with heavy metal tolerance and accumulation processes (Pereira et al., 2016).

### 4.3 Effect on photosynthesis

Photosynthesis is a well-organized and sequential process involving many components such as photosynthetic pigments, the electron transport system, and CO_2_ reduction pathways that are essential to all green plants and microorganisms. Any impairment at any of these steps has a significant impact on total photosynthetic capability (Parmar et al., 2013). Changes in pigment content are connected to visual signs of plant sickness and photosynthetic output, hence plant pigments like Chl a, Chl b, and carotenoid concentration are frequently evaluated in plants to determine the influence of environmental stress (Dresler et al., 2014). The role of Cd in the inhibition of chlorophyll biosynthesis, breakdown of pigments or their precursors, and destruction of the chloroplast membrane by lipid peroxidation due to lack of antioxidants or an increase in peroxidase activity could all contribute to a decrease in total photosynthetic pigment content (Mishra et al., 2014). Cadmium-induced reduction of photosynthetic pigments like Chl a, Chl b, and total chlorophyll has been reported in various medicinal plants like *Drimia elata* (Okem et al., 2015), *Brassica juncea* L. (Ahmad et al., 2015), *Amaranthus spinosus* (Huang J et al., 2019, Huang Y et al., 2019). Decreased performance of photosynthetic enzymes like carbonic anhydrase and RUBISCO under Cd toxicity has also been reported (Mobin and Khan, 2007; Gill et al., 2012; Parmar et al., 2013; Zaid et al., 2020). Exogenous applications of certain organic acids can reduce the phytotoxic effects of heavy metals (Hawrylak-Nowak et al., 2015; Zaheer et al., 2015). Studies revealed the application of citric acid (Mahmud et al., 2018), and salicylic acid (Krantev et al., 2008; Zhang et al., 2015) to restore the pigment content to a significant level. The effect of Cd on photosynthesis has been tabulated in **Table 3**.

**Table 3:** Changes in photosynthetic pigments and biochemical parameters in cadmium treated medicinal plants.

Plant name	Concentration and duration	Total protein and carbohydrate	Plant pigments	Lipid peroxidation	Proline	Secondary metabolites	Reference
*Adhatoda vasica* L.	100 - 600 ppm for 180 days	Decreased reducing and non-reducing sugar and total protein content	Decreased Chl a, Chl b, total chlorophyll and carotenoids with increasing concentration	–	Reduction in proline content	Reduced total alkaloids, vasicine and vasicinone	(Trivedi, 2003)
*Alternanthera tanella* Colla	50 -150 ppm for 30 days	–	Significant reduction in chlorophyll pigments	Increased at highest concentration	–	–	(Rodrigues et al., 2017)
*Artemisia annua* L.	20, 60 and 100 ppm for 336 days	–	Decreasing chl a, chl b, carotenoids and total chl with increasing concentrations and duration	Insignificant variations in MDA content between control plants and treated plants	–	Increased artemisinin production	(Li et al., 2012)
*Bacopa monnieri* L.	10- 200 ppm for 144 hours	Decreasing total protein content with increasing concentration	–	Increased lipid peroxidation	–	–	(Mishra et al., 2006; Singh et al., 2006)
*Bidens pilosa* L.	2.57ppm, 7.94 ppm, 17.33ppmand 37.17ppm for 40 days	–	Chl a, chl b and carotenoid content decreasing with increasing concentration	MDA content increased with increasing concentration	–	–	(Dai et al., 2021)
*Brassica juncea* L.	0.5 and 1 mM for 3 days	Decreased protein content	–	Increased MDA content	Enhanced production of proline	–	(Bauddh and Singh, 2012; Mahmud et al., 2018)
	200 and 300 ppm	Reduction in protein content	Reduction in chl a, chl b and total chlorophyll content	MDA content decreased	The proline content increased	–	(Ahmad et al., 2015)
*Catharanthus roseus* var. *rosea* L.	0-1000 µM for 180 days	–	The Chlorophyll content sharply reduced	MDA concentration increased with increasing concentration	–	–	(Pandey et al., 2007)
Centella asiatica (L.) Urb.	5 - 200 ppm for 30 days	–	Reduced amounts of chl a, chl b, and total carotenoids	–	–	Increased total phenolics and flavonoids and increasing centelloside concentrations with increasing concentration	(Biswas et al., 2020)
*Drimia elata* Jacq. ex Willd	2,5,10 ppm for 6 weeks	–	Significant reduction on chlorophyll a, chl b and total chlorophyll	–	Increased proline content in shoots	Decreased total phenolic and flavonoid content	(Okem et al., 2015)
*Lemna gibba* L., *Lemna minor* L.	0.01 - 1.5ppm for 96 hours	Reduced total protein content	Increased chl a, chl b, and total chlorophyll content at lower concentrations, but decreased at high concentration	Decreased MDAcontent indicating low lipid peroxidation	–	–	(Banu Doğanlar, 2013)
*Lepidium sativum* L.	20, 50 and 100 ppm for 30 days	–	Significant reduction in chl a, chl b, total chl with increasing concentration. Reduced CA activity	Increased TBARS content	–	–	(Gill et al., 2012)
*Mentha arvensis* L.	50ppm for 100 days	–	Decreased RuBisCO and carbonic anhydrase activity, decreased photosynthetic rate	Increased TBARS content	Increased proline content	–	(Zaid et al., 2020)
*Melissa officinalis* L.	0, 10, 20, and 40 mM	–	Chlorophyll a and b reduced significantly and total chlorophyll decreased at 40 mM	Malondialdehyde content increased	–	Increasing Cd conc. had a positive effect on phenolic effect with highest of ⅓ folds increase at 40mM	(Nourbakhsh Rezaei et al., 2019)
*Moringa oleifera* Lam.	1- 5 mM for 30 days	Decreasing protein content with increasing concentrations	–	–	Increasing proline content with increasing concentration	Increasing polyphenols with increasing concentrations	(Srivastava and Yadav, 2017)
*Ocimum canum* Sims.	50 - 250 mg kg^-1^for 120 days	High non reducing sugar but less amounts of reducing sugar and total protein content	Decreased amounts of Chl a, Chl b, total Chl content and carotenoids with increasing concentration	–	Enhanced proline accumulation	–	(Patel, 2006b)
*Phyllanthus amarus* Schumach. And Thonn.	10 - 100 ppm for 60 days	–	–	–	Increasing proline content along with increasing concentrations upto 50 ppm, after that a sudden decline in proline content	Decreased alkaloids, tannin and flavonoid content with increasing concentration and duration	(Dwivedi et al., 2013)
*Ricinus communis* L.	25-150 ppm for 60 days	Decreased protein content	–	Increased MDA content	Increased proline content	–	(Bauddh and Singh, 2012)
*Salvia sclarea* L.	0- 100 ppm for 8 days	–	Decreased chl a, chl b and chl a/b ratio. Increased anthocyanin content	–	–	Increased phenolics content	(Dobrikova et al., 2021)
*Satureja hortensis* L.	2.5-15mg L^−1^ for 2 weeks	Increasing soluble and reducing sugars in both roots and shoots	Reduction of chl a, chl b and total chl but anthocyanin production increased with increasing concentration	–	Enhanced proline production	High anthocyanin content, increased essential oil production	(Azizollahi et al., 2019)
*Solanum nigrum* L.	50 and 200 ppm for 3 days	Increased protein thiol content	–	Significantly high TBRAS content	–	–	(Deng et al., 2010)
*Trigonella foenum-graecum* L.	0.5 - 10mM for 30 days	–	–	Increased MDA content	–	Increased total phenolic and flavonoid content	(Zayneb et al., 2015)

### 4.4 Effect on membrane structure

The ROS emerges as a response to Cd toxicity plays an important role in the removal of hydrogen from unsaturated fatty acids, and causes severe lipid peroxidation, resulting in the production of lipid radicals and reactive aldehydes. This sets off a cascade reaction that causes lipid bilayer and membrane protein deformation (Logani and Davies, 1980). MDA is the end product of membrane lipid peroxidation, and it might indicate the extent of cell membrane damage induced by oxygen free radicals (Zhang et al., 2007). Enhanced MDA production in Cd treated plants like *B. juncea* (Bauddh and Singh, 2012; Mahmud et al., 2018), *Ricinus communis* (Bauddh and Singh, 2012; Mahmud et al., 2018), *Hibiscus cannabinus* (Li et al., 2013), *Bacopa monnieri* (Mishra et al., 2006; Singh et al., 2006) shows the severity of membrane damage under Cd stress. Decreased MDA content, as reported in *Lemna gibba* (Banu Doğanlar, 2013) can be considered as an indication of low peroxidation of lipids which can be caused by increased activity of antioxidants (Zhang et al., 2007). The antioxidant systems in plants can protect bio membranes from oxidative damage by lipid peroxidation.

### 4.5 Important biochemical changes

At lower metal concentrations and durations, an increase in protein level may be attributed to the induction of stress proteins. Stress proteins constitute various antioxidant enzymes and other enzymes involved in GSH (Glutathione) and PC (Phytochelatin) biosynthesis, including some heat shock proteins (Mishra et al., 2006). However, at higher metal concentrations, there was a significant decrease in protein content, which may be due to the Cd-induced oxidation of proteins, mediated by H_2_O_2_ and due to increased proteolytic activity (Pena et al., 2006). Proteolytic activity and protein degradation have been proposed as an index of oxidative stress (Romero-Puertas et al., 2002).

Sugars help to remove free radicals produced during stressful situations; hence their increased synthesis helps to defend against stress. These regulate osmotic potential, participate in redox processes, and aid in the maintenance of macromolecule and membrane structures (Kapoor et al., 2016). Enhanced soluble and reducing sugar has been reported in *Satureja hortensis* (Azizollahi et al., 2019), and *Withania somnifera* (Mishra et al., 2014) as a response to Cd treatment.

Total phenolic content as a surrogate measure can be used to assess antioxidative activity directly or indirectly; this is owing to their redox characteristics (Ali et al., 2007). Phenolics might play a significant role in the H_2_O_2_ detoxification process by acting as metal chelators. Flavonoids can also act as an antioxidant and have chelating properties due to their structure and substitution pattern of hydroxyl groups (Mishra et al., 2014). Enhanced production of phenolics and flavonoids, and other specific metabolites in response to Cd treatment in previous studies has demonstrated the influence of Cd on SMs (Okem et al., 2015; Srivastava and Yadav, 2017).

**Figure 2:**
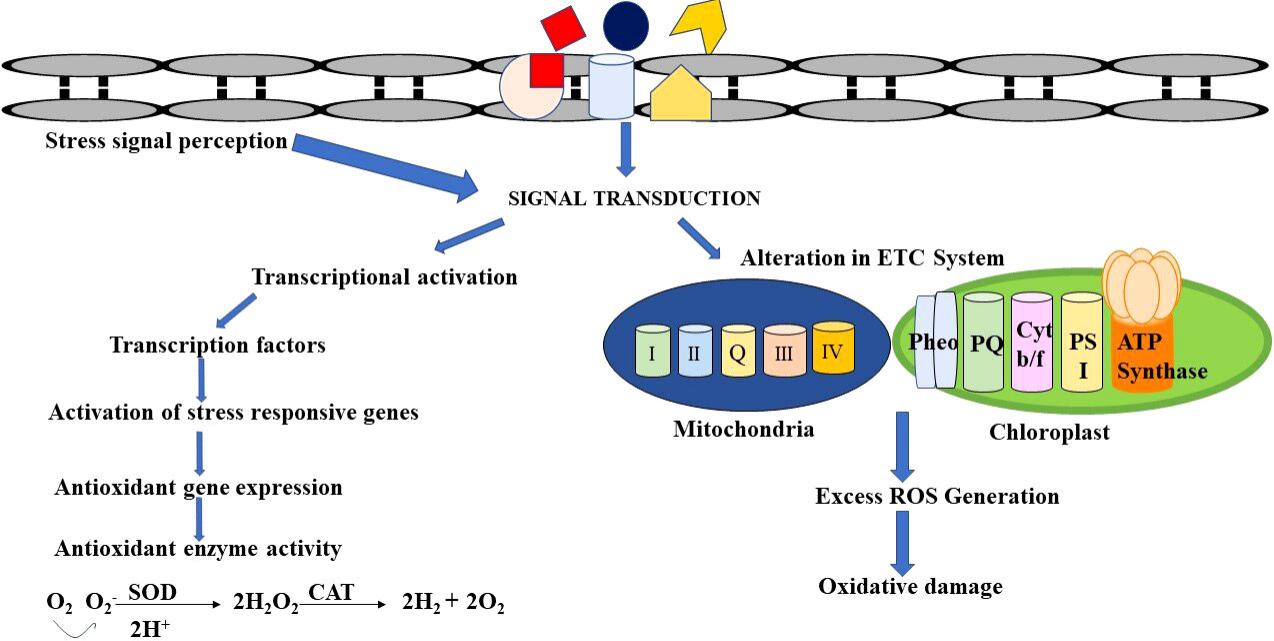
Schematic representation of defense mechanisms to counteract heavy metal stress.

Cadmium seems to be the most potent heavy metal for promoting proline synthesis (Alia and Saradhi, 1991). Proline has the ability to preserve photosynthetic equipment, electron transport complexes, membranes, enzymes, and nucleic acids by scavenging ROS (Iqbal et al., 2016; Sharmila et al., 2017). By detoxifying ROS, boosting GSH levels, and preserving antioxidative enzyme activity, proline helped to increase Cd absorption and alleviate toxicity in *Solanum nigrum* (Xu et al., 2009). Proline accumulation is also reported in plants like *Withania somnifera* (Mishra et al., 2014), *Moringa oleifera* (Srivastava and Yadav, 2017), and *Mentha arvensis* (Zaid et al., 2020). The defense mechanism to alleviate metal stress is shown in **Figure 2**.

The heavy metals that contaminate medicinal plants induce a stress response mechanism that alters the overall growth, biochemistry, and molecular status of the cell. Heavy metals can alter the efficacy of the production of SMs depending on the plant species. The biochemical changes induced by Cd in various plants are tabulated in **Table 3**. The external metal stress induces oxidative stress, which in turn triggers signal transduction and its transmission into the cell thereby altering the biosynthesis of specific plant metabolites. The ROS produced during the oxidative stress in response to heavy metal stress causes lipid peroxidation, which stimulates the production of active signaling compounds. The signaling molecules initiate or suppress the production of SMs in turn enhancing the medicinal value of the plant (Nasim and Dhir, 2010; Asgari-Lajayer et al., 2017).

**Figure 3:**
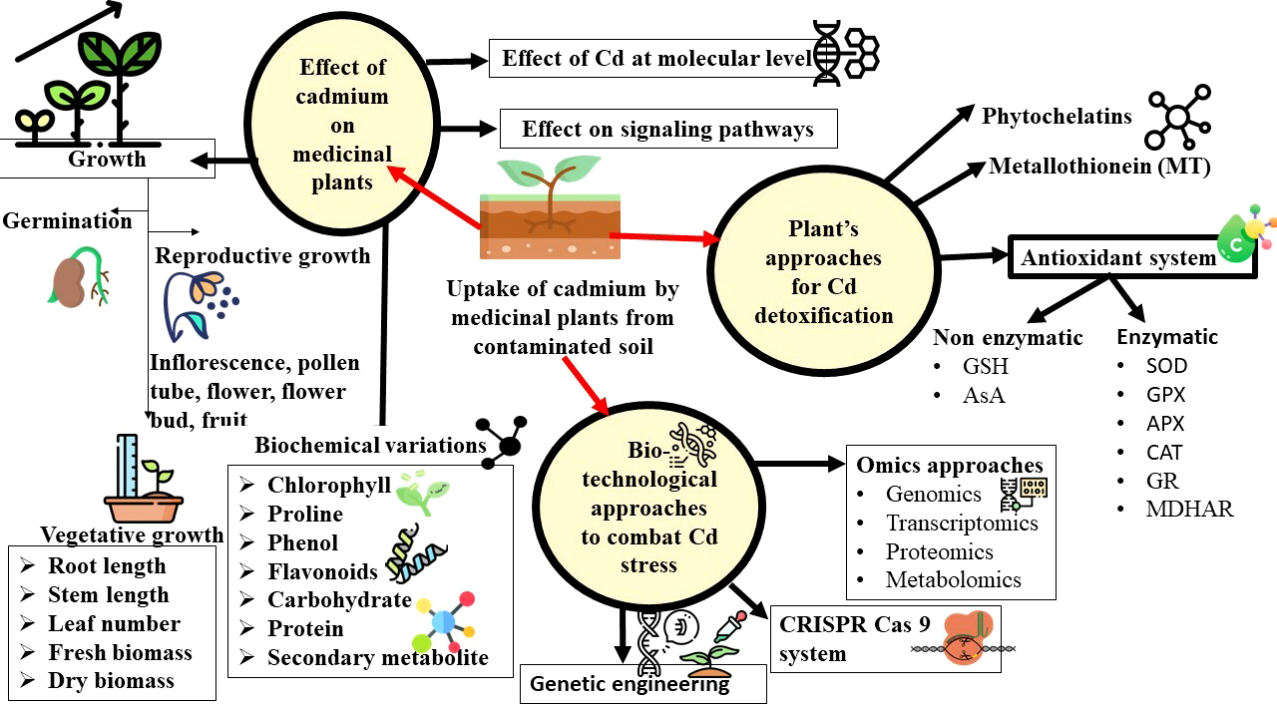
Schematic illustration of the effect of cadmium on morphological, physiological, and biochemical characteristics of medicinal plants and biotechnological and omics approaches to alleviate the cadmium stress.

In *Satureja hortensis*, low Cd concentrations elevated the levels of ɑ-terpinene, ɑ-thujene, β-phellandrene, and Ɣ-terpinene. Meanwhile, the fraction of this component decreased at high concentrations (Azizollahi et al., 2019). Similarly in *Artemisia annua*, an elevated artemisinin content was observed as a response to Cd during the initial exposure as a result of the high conversion rate of dihydroartemisinic acid to artemisinin brought by the oxygen radicals. A decline in artemisinin content was observed at 336 hours due to the enhanced toxic effect of Cd for a long duration (Li et al., 2012). The enhanced production of centelloside in *Centella asiatica* is accompanied by the overexpression of its biosynthetic genes i.e, SQS (Squalene synthase), BAS (β amyrin synthase), and CAS (cycloartenol synthase) in response to Cd treatment at high concentrations is evidence to the toxicity of Cd at the molecular level (Biswas et al., 2020). Similar results were also observed in *Phyllanthus amarus*, in which the accelerated production of phyllanthin and hypophyllanthin in presence of Cd in media, and a reduction in the production of these bioactive components at the high range of Cd exposure (Rai et al., 2005). However, in *Matricaria chamomilla*, the SMs Herniarin and Umbelliferone are unaltered by Cd treatment, while the precursors (Z)-and (E)-2-b-D glucopyranosyl oxy-4-methoxy cinnamic acids (GMCAs) increased in all the Cd concentrations (Kováčik et al., 2006). The morphological, physiological and biochemical changes in plants that are exposed to Cd stress has been represented in **Figure 3**.

### 4.6 Accumulation and translocation of heavy metal in medicinal plants

The insoluble heavy metals like Cd remain in the soil for a long time and pose a serious environmental threat. The metals enter the medicinal plants through the root system *via* channel or carrier proteins. The plant roots establish the rhizosphere in the soil by extending their root system. The roots can then accumulate and translocate specific heavy meals to shoot across cellular membranes (DalCorso et al., 2019). It is observed that generally, roots tend to accumulate heavy metals at higher concentrations when compared to stem and leaves (Zhao and Duo, 2015). The bioaccumulation studies conducted in various medicinal plants are represented in **Table 4**. Roots serve as the first line of defense, protecting other parts of the plant from metal toxicity. However, some studies report the presence of higher metal concentrations in the leaf and stem than in the root.

**Table 4:** Bioaccumulation of cadmium in different parts of medicinal plants.

Plant name	Metal Concentration	Accumulation and translocation	Reference
*Adhatoda vasica* L.	0,100, 200, 300, 400, 500, 600 ppm	Cd accumulation increased with the amount of application	(Trivedi, 2003)
*Alternanthera tanella* Colla	50- 150 ppm for 30 days	Cd absorption increased with increasing cdamium concentration	(Rodrigues et al., 2017)
*Amaranthus spinosus* L.	5-50 ppm for 60 days	The metal accumulation increased with increasing Cd concentration	(Huang Y et al., 2019)
*Andrographis paniculata* (Burm.f.) Nees	10, 50,100,150 and 200 ppm	Accumulation was high at the highest metal concentration supplemented	(Patel, 2006a)
*Bacopa monnieri* L.	5,10,50,100 *μ*M for 6 weeks	Cd accumulation increased with increasing concentration and duration with highest at 100 *μ*M concentrations	(Gupta et al., 2014)
*Cannabis sativa L.*	82, 115, 139 μg g ^-1^	The metal accumulated in roots and translocated partially to shoots	(Citterio et al., 2003)
*Centella asiatica* (L.) Urb.	50-100 ppm for 30 days	Cd accumulation was higher in treated plants than in control	(Biswas et al., 2020)
*Duranta erecta*	0.1M, 0.5M and 1M s	The plant could not tolerate the Cd stress	(Anarado et al., 2018)
*Drimia elata* Jacq. ex Willd	2, 5, 10 mg L^−1^	Accumulation of Cd increased with increasing concentrations. The bulb accumulated higher Cd concentration than shoots	(Okem et al., 2015)
*Merwilla plumbea* (Lindl.) Speta	1,5 ppm	The root accumulated the highest Cd concentration and increased significantly with concentration	(Lux et al., 2011a)
*Moringa oleifera* Lam.	1- 5 mM for 30 days	Uptake of Cd was maximum in roots and increased with increasing time interval and Cd concentration	(Srivastava and Yadav, 2017)
*Ocimum basilicum* L	0, 5 and 10 mg L^−1^ (5 days)	The Cd accumulation was higher in young plants than mature plants in their shoot and increased with increasing concentration	(Souri et al., 2019)
*Ocimum canum* Sims.	50, 100, 150, 200, 250 mg	The accumulation of Cd was dependent on the concentration of Cd treated	(Patel, 2006b)
*Sida acuta* Burm. f.	0.1M, 0.5M and 1M	Highest Cd concentration of 6.495 mg kg^-1^ was absorbed and was unable to tolerate further cadmium stress.	(Anarado et al., 2018)

## 5 Effects of cadmium on signaling pathways

The sensing and processing of signals in response to stress are key components of the plant Cd defense system and can result in certain physiologic, metabolic, and gene expression responses. In general, when the plant is exposed to stress, the plant cells activate certain genes through complex signal transduction pathways like the MAPK pathway involving phosphokinase-mediated phosphorylation and dephosphorylation reactions. A similar cascade of phosphorylation was believed to be associated with Cd signaling to the nucleus for the activation of Cd response genes. Additionally, reduced glutathione-oxidized glutathione ratio (GSH/GSSG) and elevated Calcium levels intended for the Ca-calmodulin signaling pathway were also discovered to be implicated in Cd sensing (DalCorso et al., 2010). A recent study on duckweed also supports the calcium mediated signaling of Cd (Yang et al., 2020). Apart from this, phytohormones also play an important role in Cd signaling. ABA is one such phytohormone which is actively involved in sensing Cd and subsequent response mechanisms by activating Cd defense-related genes like HMA2 and HMA4 (metal transporters) (Lu et al., 2020).

## 6 Effect of Cd at the molecular level

Under heavy metal stress, it has been discovered that transcriptional factors such as the MYB and WRKY family are triggered. OBF5, a transcription factor belonging to the bZIP family, also has been implicated in order to control the production of glutathione S-transferase (GST6) by binding to it during Cd stress thereby protecting the tissues against oxidative stress (Ghosh and Roy, 2019). In *B. juncea*, the orthologue of TGA3 protein, BjCdR15 has been observed as crucial in regulating the expression of phytochelatins and metal transporter genes (Fusco et al., 2005) and thereby taking part in cadmium uptake and long-distance transport from root to shoot. In the study by Yuan et al. (2018), the association of four transcriptional factors MYB, WRKY, ERF and bZIP family for Cd tolerance has been reported in *Agrostis stolonifera*. MYB binds to the promoter region and regulates the expression of the basic helix-loop-helix transcription factors leading to activation of IRT1, which in turn encodes a metal transporter involved in Cd uptake (Zhang et al., 2019). ERF is a transcription factor that binds and regulates MRE containing Cd stress related genes which relieves lipid peroxidation and reduces Cd accumulation (Wang et al., 2023). According to Cai et al. (2020), the TF GmWRKY142 in *Glycine max* directly affects Cd tolerance by binding to W box elements in the promoter region of cadmium stress-responsive genes GmCDT1-1 and GmCDT1-2 which decreases Cd uptake and enhances Cd resistance. The recent study in *Populus × canadensis ‘Neva’* also reported the upregulated WRKY and NAC family of TF (Li et al., 2021b). Up-regulation of *CaWRKY41* was reported in pepper as a response to Cd stress (Dang et al., 2019).

Epigenetic pathways like DNA methylation and histone modifications have become a significant, intricate aspect in how plants react to heavy metal stressors. However, only a small amount of research has emphasized how epigenetic pathways can enhance plant performance when exposed to Cd stress (Niekerk et al., 2021). When heavy metals burden plants, a key gene regulatory mechanism for plants to respond to stress and minimize toxicity is the change in methylation status in the promoter region (Chakrabarti and Mukherjee, 2021). By raising DNA methylation levels and methylating specific gene loci, plants can inhibit the production of certain genes. Alternatively, it’s possible that some genomic DNA loci are demethylated, which causes the production of specific genes and increases the stress resistance of plants (Ding et al., 2019). The hypermethylation was reported in *P. oceanica* (Greco et al., 2012) and *A. thaliana* (Li et al., 2015), while re-methylation was reported in *Raphnus sativus* (Yang et al., 2007) as a response to Cd treatment.

Apart from the epigenetic changes, Cd has been reported as a potential mutagen, which causes DNA damages including single and double-strand breaks which ultimately leads to chromosome aberrations (Ghosh and Roy, 2019). The genotoxicity of different concentrations of Cd on *Pisum sativum* was elucidated by ISSR analysis (Almuwayhi, 2021) and in *Eruca sativa* by RAPD analysis (Al-Qurainy et al., 2010). In *Catharanthus roseus*, the expression of terpenoid indole alkaloid (TIA) genes like STR, DAT, SGD, SLS, PRS, TDC, MTs, TPT, and MDR has been triggered under Cd toxicity (Chen et al., 2018c). In the comparative study of Cd-sensitive and Cd-tolerant *Medicago truncatula*, it was found that the overexpression of GSH and phytochelatin biosynthesis genes such as MtCYS, MtγECS, MtGSHS, MtGR, and MtG6PDH in Cd sensitive plant (García de la Torre et al., 2022). While the enhanced expression of metallothionein genes from the families MT1, MT2 and MT3 in response to Cd stress in *Phytolacca americana* was reported by Chen et al. (2017). In addition, the activation of Potri.010G183900 (ABA gene) was observed in *Populus × canadensis ‘Neva’* as a response to Cd exposure (Li et al., 2021b).

## 7 Plant’s approaches for cadmium detoxification

On exposure to heavy metals, plants try to avoid or minimize absorption into root cells by binding metal ions to the cell wall or cellular exudates or by limiting them to the apoplast or reducing long-distance transport as the first line of defense. Releasing of some root exudates or other low molecular weight substances to minimize the effect of Cd, like the release` of lubimin and 3-hydroxylubimin in response to Cd toxicity in *Datura stramonium* root cultures, is a kind of such defense (Furze et al., 1991). When metal ions are present at high levels, cells engage an array of detoxification and storage methods, including chelation of metal ions in the cytosol with phytochelatins and metallothioneins, trafficking, and sequestration into the vacuole *via* vacuolar transporters (Zhou et al., 2015). The presence of Cd as electron-dense granules in both cell wall and cytoplasmic compartments of root and vacuoles of spongy and palisade parenchyma cells in leaves of *Thlaspi caerulescens* along with enhanced phytochelatin production might be due to the Cd-phytochelatin complexation followed by compartmentalization (Wójcik et al., 2005).

Cadmium can trigger the generation of phytochelatins, which are tiny metal-binding peptides (PCs) that have the basic structure (-Glu-Cys)n-Gly, with n = 2–11. The PCs bind Cd and form varied complexes with molecular weights of around 2,500–3,600Da. The Cys thiolic groups of PC guard the cytosol from free Cd ions and eventually sequester Cd in the vacuole. The synthesis of PCs from Glutathione is catalyzed by the cytosolic PC synthetase (Cobbett and Goldsbrough, 2002; Ahmad et al., 2019). Various studies on medicinal plants like *Bacopa monnieri* (Mishra et al., 2006; Singh et al., 2006), *Solanum nigrum* (Deng et al., 2010), *Thlaspi caerulescens* (Wójcik et al., 2005), *B. juncea* (Mahmud et al., 2018) reveals the role of phytochelatins in reducing the effects of Cd toxicity.

Reduced glutathione (GSH) is a glutamic acid, cysteine, and glycine amino acid derivative. It can be used as a ligand to chelate heavy metals, and so reduce their toxicity (Yu et al., 2019). GSH is known to alleviate Cd-induced oxidative stress by positively controlling the activities of antioxidant enzymes and the expression of transcription factors involved in the regulation of stress response genes (Hasan et al., 2016). Enhanced GSH production is reported in *Bacopa monnieri* (Mishra et al., 2006; Singh et al., 2006), *Lepidium sativum* (Gill et al., 2012), *Solanum nigrum* (Deng et al., 2010) with increasing Cd concentrations along with the increased antioxidant enzymes.

Metallothionein (MT) is a cysteine-rich, metal-binding protein (Yu et al., 2019) that chelates metal ions and forms MT–metal complexes and tend to be found in the cytosolic compartments. MTs, apart from PCs, are the outcome of mRNA translation associated with heavy metal stress (Cobbett and Goldsbrough, 2002; Ahmad et al., 2019). Increased amounts of metallothionein in response to high concentrations of Cd in plants like *Moringa oleifera* (Srivastava and Yadav, 2017) indicate its importance in Cd detoxification.

The toxic effects of ROS may be alleviated either by non-enzymatic (GSH; ascorbic acid, ASA;-tocopherol and carotenoids) or by enzymatic SOD (superoxide dismutase), CAT (catalase), APX (ascorbate peroxidase), GR (glutathione reductase), MDHAR (monodehydroascorbate reductase dehydroascorbate) antioxidants. Antioxidant enzymes play a crucial role in diminishing the adverse effects of reactive oxygen species formed under Cd stress to improve plant growth and metabolic tolerance (Luo et al., 2011; Biswas et al., 2020).

SOD is an important part of the antioxidative defense machinery, which helps to exclude superoxide radicals, reduce the peroxidation of membrane lipids and retain the stability of the cell membrane (Zhang et al., 2007). The reduction in the SOD activity under a high dose of Cd stress as reported in *Alternanthera tanella* (Rodrigues et al., 2017), *Withania somnifera* (Mishra et al., 2014), and *Hibiscus cannabinus*, might be attributed to enzyme damage due to the excessive production of free radicals and peroxides (Mishra et al., 2006; Li et al., 2013). The SOD converts O_2_^–^ to H_2_O_2_ and efficiently blocks O_2_^–^ driven cell damage. Since a sheer volume of H_2_O_2_ limits the plant’s capacity to tolerate Cd, the oxidoreductase enzymes CAT, APX, GPX, and POD work together to prevent H_2_O_2_ buildup (Raza et al., 2020) by reducing them into the water and molecular oxygen by working at different locations in the cell. APX functions in chloroplasts in the ascorbate–glutathione cycle, whereas GPX is basically a cell wall-bound enzyme and is also found in cytoplasm while CAT is present in peroxisomes and mitochondria (Mishra et al., 2006).

According to Li et al. (2013) and Mishra et al. (2006), the reduced CAT activity observed in Cd treated *Bacopa monnieri* plants in both leaves and roots may be attributed to degradation caused by increased peroxisomal proteases, photoinactivation of the enzyme, or inactivation owing to excessive oxygen radicals. A similar reduction of CAT activity was also reported in *Trigonella foenum-graecum* (Zayneb et al., 2015) and *B. juncea* (Mahmud et al., 2018). The enhanced activity of the other two H_2_O_2_ degrading enzymes, APX and GPX or POD, appears to have compensated for the reduced activity of CAT in all these plants. The cadmium detoxification approaches undertaken by plants are represented in **Table 5**.

**Table 5:** Defense mechanisms for Cadmium tolerance in medicinal plants.

Plant	Concentration and duration	Chelating agents/detoxification proteins(Phytochelatins and metallothioneins, reduced GSH etc)	Antioxidant enzymes(SOD, CAT etc)	Reference
*Alternanthera tanella* Colla	50- 150 ppm for 30 days	–	Decreased SOD activity at high concentration and increased APX activity	(Rodrigues et al., 2017)
*Bacopa monnieri* L.	10- 200 ppm for 144 hours	Enhanced production of phytochelatins along with GSH production.	Enhanced APX and GPX activity but reduced CAT activity with increasing concentration	(Mishra et al., 2006; Singh et al., 2006)
*Bidens pilosa* L.	2.57ppm, 7.94 ppm, 17.33ppm, and 37.17ppm for 40 days	–	SOD, GPX and GR activity increased with metal concentration	(Dai et al., 2021)
*Brassica juncea* L.	0.5 and 1mM for 3 days	Enhanced phytochelatin production. GSH content increased at lower concentration, but decreased at high concentration	Increased GPX and SOD activity, reduction in CAT activity at high concentration	(Mahmud et al., 2018)
	200 and 300 ppm	–	SOD, CAT, APX and GR activity increased with increasing concentration	(Ahmad et al., 2015)
*Cajanus cajan* L.	10-30 ppm	–	CAT and POD increased in leaves, root and stem of seedling	(Patel and Patel, 2012)
*Cannabis sativa* L.	82, 115, 139 μg g^−1^	An increase in the PC and GSH was observed	–	(Citterio et al., 2003)
	27 and 82 ppm	Increased GSH and phytochelatin content	–	(Citterio et al., 2003)
*Centella asiatica* L.	5 - 200 ppm for 30 days	–	Enhanced SOD, GPX and APX activity	(Biswas et al., 2020)
*Hibiscus cannabinus* L.	20-120 ppm	High GSH content at lower concentrations but decreased at high concentration in both leaves and roots	SOD, CAT and POD activities in roots increased and then dropped at high concentration	(Li et al., 2013)
*Lemna gibba* L., *Lemna minor* L.	0.01 - 1.5ppm for 96 hours	–	Increased activities of CAT, APX and POD	(Banu Doğanlar, 2013)
*Lepidium sativum* L.	20, 50 and 100 ppm for 30 days	High GSH content	Increased activities of SOD, APX, CAT and GR	(Gill et al., 2012)
*Melissa officinalis* L.	0.10, 20, and 40 mM	–	CAT and SOD activities increased	(Nourbakhsh Rezaei et al., 2019)
*Mentha arvensis* L.	50ppm for 100 days	–	Enhanced activities of SOD, CAT, POX, and GR	(Zaid et al., 2020)
*Moringa oleifera* Lam.	1- 5 mM for 30 days	Significant increase in metallothionein concentration	Increased CAT, APX and GR activity	(Srivastava and Yadav, 2017)
*Phyllanthus amarus* Schumach. and Thonn.	10-100 ppm for 60 days	–	Increased CAT and APX activity upto 30 - 60 ppm. Sudden decline at higher concentrations	(Dwivedi et al., 2013)
*Satureja hortensis* L.	2.5- 15ppm	–	Enhanced CAT and APX activity	(Azizollahi et al., 2019)
*Solanum nigrum* L.	50 and 200 ppm for 3 days	Increased phytochelatin production and High GSH content	Enhanced activities of SOD, APX, GR, CAT, POD And GSH-PX activity	(Deng et al., 2010)
*Thlaspi caerulescens* J. and C. Presl	500 ppm for 9 weeks	Increased phytochelatin content	–	(Wójcik et al., 2005)
*Trigonella foenum-graecum* L.	0.5 – 10 mM for 30 days	–	Increasing SOD and APX activity with increasing concentrations in both roots and shoots but, reduced activity of CAT with high concentrations	(Zayneb et al., 2015)
*Withania somnifera* L. Dunal.	5 - 1000ppm for 30 days	–	Increased activities of CAT, G -POD, POD, GPX, APX, reduced activity of SOD and GR at high concentrations	(Mishra et al., 2014)

## 8 Various approaches to ameliorate heavy metal stress in medicinal plants

### 8.1 Omics approaches

Deciphering the actual mechanisms by which heavy metals induce stress and understanding the physiological, biochemical, and molecular responses to metal toxicity at the cellular level is an extremely hard and challenging task. Thus, over the last few years, modern biotechnological tools are employed to understand the mechanisms underlying plant-metal interaction. In this section, we will provide a unique perspective of metal-induced toxicity and its reclamation by regulation of proteomics, metabolomics, and epigenomics changes in plants. Omics approaches such as genomics, transcriptomics, miRNAomics, proteomics, metabolomics including metallomics, are pragmatic approaches that provide a complete understanding of physiological, biochemical, and molecular responses to stress in plants (Rai et al., 2021) and can be used to develop stress tolerant and resilience plant systems (Jamla et al., 2021). The various omics approach study conducted in medicinal plants is represented in **Table 6**.

**Table 6:** Omics approaches for understanding response to cadmium stress in medicinal plants.

Omics approach	Plant	Method	Outcome of study	Reference
Genomics	*Brassica napa* L.	TILLING	role of the HMA4 gene has been elucidated	(Navarro-León et al., 2019)
Genomics	*Brassica napa* L.	GWAS	Identified NRAMP6 (natural resistance-associated macrophage protein 6), IRT1 (iron-regulated transporter 1), CAD1 (cadmium-sensitive 1), and PCS2 (phytochelatin synthase 2) genes as candidate genes for Cd accumulation	(Chen et al., 2018b)
Genomics	*Linum usitatissimum* L.	GWAS	Identified 198 ABC transporters and 12 HMA gene	(Khan et al., 2020)
Genomics	*Medicago sativa* L.	GWAS	genes such as oxidative stress response genes, P type transporters genes are associated with Cd tolerance	(Paape et al., 2021)
Genomics	*Oryza sativa* L.	CRISPR Cas 9	Role of OsNramp1, OsNramp5, OsLCT1 gene to reduce Cd uptake	(Wang et al., 2019; Songmei et al., 2019; Chen et al., 2019)
Genomics	*Solanum tuberosum* L.	qRT-PCR analysis	Identification of 11 MTP genes from Mn-MTP, Zn-MTP and Zn/Fe-MTP gene families	(Li et al., 2021a)
Transcriptomics	*Brassica napus* L.	RT-PCR	Identification of 13 conserved miRNAs involved in response mechanism to Cd stress	(Huang et al., 2010)
Transcriptomics	*Brassica napus* L.	high-throughput sequencing	Identification of 44 known miRNAs (belonging to 27 families) and 103 novel miRNAs involved in Cd stress response	(Jian et al., 2018)
Transcriptomics	*Cucumis sativus* L.	RNA sequencing	Identification of transporter genes (CsHMA1, CsNRAMP1, CsNRAMP4, CsZIP1, and CsZIP8)	(Feng et al., 2021)
Metabolomics	*Amaranthus hypochondriacus* L.	LC MS	purine metabolism, Gly, Ser, and Thr metabolism, as well as Pro and Arg metabolism, are all involved in the improved tolerance at the vegetative stage	(Mengdi et al., 2020)
Metabolomics	*Brassica napus* L.	HPLC	Decreased Cd accumulation in roots and shoots	(Ali et al., 2020)
Metabolomics	*Catheranthus roseus* var. *rosea* L.	GC MS	differential accumulation of secondary metabolites was found to be responsible for the cd tolerance	(Rani et al., 2021)
Metabolomics	*Salvia miltiorrhiza* Bunge	GC MS	Cd boosted Rosmarinic Acid production *via* controlling amino acid metabolism but hindered tanshinone synthesis primarily by decreasing the GGPP concentration, with proline, POD, and CAT playing critical roles	(Yuan et al., 2021)
Metallomics	*Arabidopsis halleri*	μ-XRF using high-energy synchrotron radiation	cellular distribution of cadmium	(Fukuda et al., 2020)
miRNAomics	*Boehmeria nivea* L. Gaudich	high-throughput sequencing and *silico* method	Identification of 73 novel miRNAs and 426 potential miRNA targets which has been involved in metal ion absorption, chlorophyll biosynthesis and protein ubiquitination	(Chen et al., 2018b)

#### 8.1.1 Genomics

Genomic approach includes the identification of genes involved in metal resistance, transport of heavy metals, and plant stress tolerance. Clustered regularly interspaced short palindromic repeats (CRISPR/Cas9), DNA mismatch repair (MMR), targeted induced local lesions in genomes (TILLING), and Genome-wide association studies (GWAS) are some of the genomic approaches to understand the genes involved in plant -metal interaction (Raza et al., 2020). In *Brassica rapa*, subjected to CdCl_2_ stress, the role of the HMA_4_ gene has been studied using targeted induced local lesions in genomes (TILLING) (Navarro-León et al., 2019). Genome-wide association studies (GWAS) have been conducted in *Brassica rapa* using a 60K Brassica Infinium^®^ SNP array to understand the mechanisms underlying Cd tolerance (Chen et al., 2018b). Another GWAS study in *Medicago sativa* subjected to cadmium stress reveals that the root and leaf response traits are polygenic with multiple quantitative loci (QTL), and genes such as oxidative stress response genes, P-type transporters genes are associated with Cd tolerance (Paape et al., 2021). Studies have been conducted in the OSNramp5 gene to reduce Cd uptake in rice by CRISPR Cas 9 technology (Wang et al., 2019). In the cadmium toxicity studies conducted by Zhao et al. (2020) in *Glycine max* (L.), it has been found that MSH2 and MSH6 of the mismatch repair system (MMR) have played an important role in tolerance to cadmium stress.

#### 8.1.2 Transcriptomics and proteomics

A group of small RNAs, such as miRNA and siRNA, are involved in post-transcriptional regulation. Moreover, a group of miRNAs is reported to be involved in responsive mechanisms to plant stress. A total of 13 conserved miRNAs involved in the response mechanism to Cd stress are identified by transcriptional analysis with RT-PCR (Huang et al., 2010), and a total of 44 known miRNAs (belonging to 27 families) and 103 novel miRNAs have been identified by high-throughput sequencing (Jian et al., 2018) in *Brassica napus* L. A total of 73 novel miRNAs (identified by high throughput sequencing), and 426 potential miRNA targets (identified by *in silico* method) are reported to be involved in metal ion absorption, chlorophyll biosynthesis, and protein ubiquitination in *Boehmeria nivea* L. (Chen et al., 2018a).

In *B. juncea* L. fifty-two genes out of seventy-three Cd responsive transcript derived fragments were identified as gene expression regulators, stress-responding transcriptional factors, and transport facilitation genes by cDNA-amplified fragment length polymorphism (cDNA-AFLP) analysis (Fusco et al., 2005). In the transcriptomics study conducted by Feng et al. (2021) five transporter genes: CsHMA1, CsNRAMP1, CsNRAMP4, CsZIP1, and CsZIP8 have been identified in *Cucumis sativus* L. and it has been observed that the transcript of CsNRAMP4 positively correlated and the expression level of CsHMA1 negatively correlated with Cd accumulation. The transcriptomics study in *Lactuca sativa* L. var. *ramose* using PacBio and Illumina techniques reveal the potential molecular pathway (antioxidant and hormone signal transduction) under Cd stress with and without pre-application of melatonin. The genes involved in Cd detoxification on melatonin application are identified (Yu et al., 2022). The transcriptomic study conducted in two different cultivars of *Brasica rapa* var chinensis (Baiyewuyueman and Kuishan’aijiaoheiye) identified 797 ROS-related proteins and 1167 transcription factors encoding unigenes. These four genes (DEGs, SOD1, POD A2/44/54/62 and GST1) are associated with the differential response to Cd stress between the two cultivars (Yu et al., 2017). Proteomics studies in *Populus yunnanensis* under cadmium stress reveal the protective role of nitrogen in alleviating cadmium stress. It was observed that 42 proteins and 522 proteins were upregulated in groups treated with cadmium along with nitrogen when compared to Cd-treated and control plants, respectively, and 89 proteins and 127 proteins were down-regulated by Cd+ N treatment in comparison to Cd-treated and control plants respectively (Huang J et al., 2019).

#### 8.1.3 Metabolomics

With the aid of metabolomics techniques, scientists may better understand the fundamental metabolite profiles that confer stress resistance in plants and generate these profiles in any crop species to increase their resilience to biotic and abiotic challenges, including climate change (Singh et al., 2021). Primary metabolism, which encompasses sugars, amino acids, and nucleic acids, influences how plants adapt to their environment, while, secondary metabolites are non-essential rather than play pleiotropic functions in modifying plant responses to abiotic and biotic stressors (Zou et al., 2022). An analysis of *Arabidopsis*s’ proteome and metabolome revealed that the main reaction of the metabolome to Cd stress was to activate the carbon, nitrogen, and sulfur metabolism, which led to the formation of Cd-chelating compounds (phytochelatins) (Sarry et al., 2006).

According to a study performed on *Amaranthus hypochondriacus*, the nine pathways responsible for antioxidation, osmotic balance regulation, energy supplementation, and the promotion of metabolites that participate in phytochelatin (PC) synthesis were the main sites of involvement for the metabolites under Cd toxicity in various growth stages (Mengdi et al., 2020). Additionally, they discovered that the purine metabolism, Gly, Ser, and Thr metabolism, as well as Pro and Arg metabolism, are all involved in improved tolerance at the vegetative stage. The most significant metabolic indicator of Cd stress in the *Amaranthus hypochondriacus* was discovered to be purine metabolism (Mengdi et al., 2020). Similar to this, a metabolomics investigation in tobacco plants under Cd stress, showed 150 and 76 metabolites, were differently deposited in the roots and leaves respectively. These metabolites were much more abundant in the production of flavone and flavonols, nicotinate and nicotinamide, arginine and proline, and amino acids (Zou et al., 2022).

However, in *Catharanthus roseus*, the differential accumulation of secondary metabolites is thought to be responsible for the Cd tolerance (Rani et al., 2021). The increased levels of metabolites from the monoterpenoid indole alkaloid pathway, including nicotine, coronaridine, vidorosine, vindoline, tabersonine, and indolinine. In addition to isoprenoids and polyamines, other responsive metabolites included terpenes such as caryophyllene, campestrin, phytol, neophytadiene, cedrol, and silicone oil, emphasizing the significance of secondary metabolites in Cd tolerance in *Catharanthus roseus* (Rani et al., 2021). Likewise, the metabolomic findings indicate that Cd boosted RA production *via* controlling amino acid metabolism but hindered tanshinone synthesis primarily by decreasing the GGPP concentration, with proline, POD, and CAT playing critical roles in *Salvia miltiorrhiza*’s capacity to endure Cd stress (Yuan et al., 2021). The reaction to Cd stress in *Calendula officinalis* plant roots appeared to be more influenced by metabolic changes, such as an increase in sterol production simultaneous to a decrease in the triterpenoid content of the plant roots and hairy root culture (Rogowska et al., 2022).

#### 8.1.4 Metallomics

Metallomics involves analytical approaches for characterizing the entirety of metal biomolecules in an organism (metallome) (Gómez Ariza et al. 2013). Metallomics, which includes the identification of metals (qualitative metallomics) and determining their levels (quantitative metallomics), may promote the development of applications for improved techniques in metal-contaminated soils (Singh and Verma 2018). To assure the safety of therapeutic plants and products, harmful metals present in them can be identified using metallomics tools such as HR-ICP-SFMS (Kenny et al. 2022). Identifying metal-binding proteins such as phytochelatins and metallothioneins can be used as biomarkers for the heavy metal stress that medicinal plants experience (Singh and Verma 2018). Fukuda et al. (2020) identified the distribution as well as accumulation of Cd in the leaves and trichomes of *Arabidopsis halleri* ssp. gemmifera using X-ray microfluorescence analysis. Likewise, ICP-MS was used to measure the Cd level in roots, stems, and early leaves. Micro XRF mapping with synchrotron radiation was also used to precisely locate Cd in various plant tissues (Pongrac et al. 2018). In addition, using XAS, the putative S- or O-based Cd ligands in the leaf tissue of several Cd-hyper-accumulating Brassicaceae species have been investigated (Jamla et al. 2021).

### 8.2 Biotechnological approaches

#### 8.2.1 Genetic engineering approaches

Hyperaccumulator plants survive heavy metal stress and show metal tolerance by active detoxification and sequestration. They gain this ability due to the presence of stress tolerance genes in them. Thus, the metal-sensitive plants can be genetically modified for metal uptake, transport, and sequestration by the transformation of the genes of metal-hyperaccumulating plants that can accumulate, translocate and detoxify metals at a faster rate (Weerakoon, 2019). An attempt to enhance the cadmium accumulation and tolerance in *Solanum nigrum L*. has been made by Ye et al. (2020). The hairy roots of *S. nigrum* were infected with *Agrobacterium rhizogenes* ATCC15834 carrying the iron-regulated transporter gene (IRT1) from *A. thaliana*. The IRT1 gene expressed in transgenic *S. nigrum* reduced the phytotoxic effects of cadmium, enhanced cadmium tolerance, and helped in the normal growth of the plant. Transgenic *Medicago truncatula* expressing Delta(1)-pyrroline-5-carboxylate synthetase (P5CS) has been established b*y* Verdoy et al. (2006) by infecting the host plant with *Agrobacterium rhizogenes* EHA105 carrying *VaP5CS* from *Vigna aconitifolia.* The *VaP5CS gene in* transgenic *Medicago truncatula* has been reported as being involved in conferring cadmium tolerance by enhanced proline accumulation and antioxidant activit*y* by García de la Torre et al., (2022). Very limited attempts at genetic engineering in medicinal plants have been reported till date. Thus, there is a need for extensive studies on genetic improvements to confer metal tolerance mechanisms in medicinal plants. The widely used gene editing approaches such zinc finger nucleases (ZFNs) and transcript activators like effector nucleases (TALENs) is limited due to the frequent mutations at non-targeted sites (Sarma et al., 2021).

#### 8.2.2 CRISPR/Cas system, a gene editing approach for heavy metal tolerance

The CRISPR–Cas9 (clustered regularly interspaced short palindromic repeats (CRISPR)/CRISPR-associated protein 9) system has emerged as an innovative gene editing tool in plant systems (Venegas-Rioseco et al., 2021). It is a quick, cost friendly tool that can be used for improving crop traits against abiotic and biotic stress tolerance (Pandita, 2021). It consists of spacer sequences placed between short palindromic repeats which transcribe to CRISPR RNA (crRNA), which combines with transactivating crRNA (tracrRNA) to form a mature crRNA/tracrRNA complex (guide RNA/gRNA). The gRNA directs the Cas nuclease, which creates a DNA double-strand break (DSB) in the desired DNA sequence, thereby causing gene deletion *via* the repair mechanisms of cells (Rai et al., 2021; Venegas-Rioseco et al., 2021). Thus, gRNA-guided–Cas9 systems can be used for gene knockout, regulation of gene expression, and transcription factors, thereby enhancing heavy metal stress tolerance and phytoremediation in a diverse range of plants (Bao et al., 2019).

The important genes involved in metal stress tolerance in phytoremediator plants such as *Arabidopsis halleri*, *B. juncea, Hirschfeldia incana, Noccaea caerulescens*, and *Pteris vittata* can be identified through transcriptomics to develop ideal hyperaccumulator plants (Kumar and Trivedi, 2018). Further, the incorporation of advanced gene editing technologies such as CRISPR–Cas9 will help enhance phytoextraction technology (Thakur et al., 2020). The Cd accumulation in *Oryza sativa* has been reduced by knocking out the OsNramp5 metal transporter gene using the CRISPR-dCas9 system (Tang et al., 2017). The gene expression can be modulated by fusing the transcription factors with dCas9 to upregulate or downregulate the expression of a gene or a group of genes of interest (Miglani, 2017). The cytoplasmic Cd has been detoxified and enhanced the cadmium tolerance in *A. thaliana* by inducing the gene expression of AtPDF2.6 (Luo et al., 2019). Thus, CRISPR–Cas9 is a promising approach for enhancing the natural capacity of a plant to grow, accumulate, and tolerate heavy metal stress without introducing foreign genes (**Figure 3**).

## 9 Conclusion and prospects

In recent years, herbal drugs have been gaining popularity. Thus, the quality of herbal-based drugs has to be guaranteed prior to their marketing. The herbal drugs must be free of heavy metal contaminants, the presence of which would otherwise suppress the growth of the medicinal plant and affect the biosynthesis of important SMs either by upregulating or downregulating the genes involved in the biosynthetic pathway of SMs. In conclusion, it has been observed that the different growth stages including germination, vegetative and reproductive growth, photosynthesis, and biochemical parameters have been affected in different medicinal plants on exposure to cadmium. Most of the medicinal plants exposed to cadmium toxicity exhibit cadmium detoxification mechanisms such as the generation of phytochelatins, and metallothioneins, and triggering non-enzymatic and enzymatic antioxidant responses. The omics technology has been adopted to understand the mechanisms underlying plant-metal interaction. However, it is evident that very few genetic engineering approach studies have been conducted to confer cadmium resistance in medicinal plants and no CRISPR Cas 9 genetic tool approach has been reported in medicinal plants except a few crop plants. As is customary, extensive research has to be conducted to elucidate the defense mechanism involved in cadmium tolerance and its detoxification. Furthermore, CRISPR Cas 9 gene editing technique has to be employed for tailoring medicinal plants against Cd stress. Genetically modified medicinal plants derived through gene editing tools have to be assessed for their reliability. There is a scope for the adoption of synthetic biology approaches to develop improved varieties with heavy metal tolerance.

Discerning the toxic concentration of heavy metals and tolerance indices of medicinal plants would be beneficial in the establishment of a high-quality environment for plant growth. With the findings on the ability of the medicinal plants to uptake, accumulate, and translocate heavy metals, it is possible to have a better management program for growing medicinal plants, its safe consumption and usage in herbal drugs.

## Author contributions

PN conceived the review. AB and RR collected the literature and wrote the manuscript. PN, helped in the original draft. PN and JMA-K critically reviewed the initial draft and streamlined the idea. AB and RR prepared and revised the tables and figures. JMA-K helped in funding acquisition and JMA-K, FMA, and MIA helped in revision of the manuscript. All authors carefully read, revised, and approved the manuscript for submission.

## Glossary

**Table:**

ABA	Abscisic acid
ATCC15834	*Agrobacterium rhizogenes* ATCC 15834
AtHMA2	*Arabidopsis thaliana* heavy metal transporting ATPase transporter protein 2
AtHMA3	*Arabidopsis thaliana* heavy metal transporting ATPase transporter protein 3
AtHMA4	*Arabidopsis thaliana* heavy metal transporting ATPase transporter protein 4
AtIRT1	*Arabidopsis thaliana* iron-regulated transporter-like proteins 1
BAS	β amyrin synthase
bZIP	Basic leucine zipper family of transcription factors
BjCdR15	*Brassica juncea* Cd resistant gene 1
CaWRKY41	Capsicum WRKY transcription factor family gene 1
Cd	Cadmium
Chl	Chlorophyll
CRISPR- Cas 9	Clustered regularly interspaced short palindromic repeats/ CRISPR associated protein 9
crRNA	CRISPR RNA
CAS	cycloartenol synthase
DEGs	Differentially expressed genes
MMR	DNA mismatch repair
ERF 1 and ERF 2	ethylene response factors 1 & 2
GWAS	Genome-wide association studies
GGPP	Geranylgeranyl diphosphate
GMCAs	glucopyranosyl oxy-4-methoxy cinnamic acids
GSH	Glutathione
GmCDT1-2	*Glycine max* Cd tolerance 1-2
GmWRKY142	*Glycine max* WRKY transcription factor family gene 142
Gly, Ser, and Thr	Glycine, serine and threonine
gRNA	Guide RNA
HMA	heavy metal transporting ATPase transporter protein
HR-ICP-SFMS	High-resolution inductively coupled plasma sector field mass spectrometry
ICP-MS	Inductively coupled plasma mass spectrometry
MDA	Malondialdehyde
MtZIP6	*Medicago truncatula* zinc-regulated transporters 6
MTPs	metal tolerance or transporter proteins
MT1, MT2 and MT3	Metallothionine 1, 2 and 3
miRNA	Micro RNA
Micro XRF	Micro X-ray fluorescence
MAPK	Mitogen-activated protein kinase
MRE	Metal responsive elements
MYB	Myeloblastosis related gene family of transcription factors
NAC, ATAF1/2	(NAM and CUC2) family of proteins
NcZNT1	*Noccaea caerulescens* zink transporter gene 1
NRAMP	Natural resistance-associated macrophage protein
OsHMA2	*Oryza sativa* heavy metal transporting ATPase transporter protein 2
OsHMA3	*Oryza sativa* heavy metal transporting ATPase transporter protein 3
OsIRT1	OsIRT2
Oryza sativa iron-regulated transporter-like proteins 1	*Oryza sativa* iron-regulated transporter-like proteins 2
OsNRAMP1, OsNRAMP5	*Oryza sativa* natural resistance-associated macrophage protein 1 and 5
OsZIP6	*Oryza sativa* zinc-regulated transporters 6
PC	Phytochelatin
Pro and Arg	Proline and Arginine
P5CS	pyrroline-5-carboxylate synthetase
QTL	quantitative loci
RAPD	Random Amplified Polymorphic DNA
GSH/GSSG	reduced glutathione-oxidized glutathione ratio
RT-PCR	Reverse transcription polymerase chain reaction
RUBISCO	Ribulose bisphosphate carboxylase/oxygenase
RA	Rosmarinic acid
DSB	double strand break
SMs	Secondary metabolites
SNP	Single nucleotide polymorphism
siRNA	Small interfering RNA
SQS	Squalene synthase
SOD1, POD	Super oxide dismutase, peroxidase
TILLING	targeted induced local lesions in genomes
TGA3	TGACG-binding (TGA) transcription factors 3
tracrRNA	transactivating crRNA
TALENs	Transcript activators like effector nucleases
TF	Transcriptional factors
WHO	World Health Organization
WRKY	WRKY transcription factor gene family
XAS	X-ray absorption spectroscopy
ZFNs	Zinc finger nucleases
ZIP	zinc-regulated transporters
ZRT-IRT-like proteins	Zink/iron-regulated transporter-like proteins

## References

[B1] Aghaz M. Bandehagh A. Aghazade E. Toorchi M. Ghassemi-Gholezani K. (2013). Effects of cadmium stress on some growth and physiological characteristics in dill (*Anethum graveolens*) ecotypes. Int. J. Agriculture 3, 409–413.

[B2] Ahmad J. Ali A. A. Baig M. A. Iqbal M. Haq I. Irfan Qureshi M. (2019). “Role of phytochelatins in cadmium stress tolerance in plants,” in Cadmium toxicity and tolerance in plants. Eds. Hasanuzzaman M. Prasad M. N. V. Fujita M. (London, UK: Academic Press), 185–212.

[B3] Ahmad S. H. Reshi Z. Ahmad J. Iqbal M. (2005). Morpho-anatomical responses of *Trigonella foenum graecum* linn. to induced cadmium and lead stress. J. Plant Biol. = Singmul Hakhoe Chi 48, 64–84. doi: 10.1016/B978-0-12-814864-8.00008-5

[B4] Ahmad P. Sarwat M. Bhat N. A. Wani M. R. Kazi A. G. Tran L.-S. P. (2015). Alleviation of cadmium toxicity in *Brassica juncea* l. (Czern. and coss.) by calcium application involves various physiological and biochemical strategies. PloS One 10, e0114571. doi: 10.1007/BF03030566 25629695 PMC4309397

[B5] Alia Saradhi P. P. (1991). Proline accumulation under heavy metal stress. J. Plant Physiol. 138, 554–558. doi: 10.1016/S0176-1617(11)80240-3

[B6] Ali M. B. Hahn E.-J. Paek K.-Y. (2007). Methyl jasmonate and salicylic acid induced oxidative stress and accumulation of phenolics in *Panax ginseng* bioreactor root suspension cultures. Molecules 12, 607–621. doi: 10.3390/12030607 17851415 PMC6149333

[B7] Ali E. Hassan Z. Irfan M. Hussain S. Rehman H.-U. -. Shah J. M.. (2020). Indigenous tocopherol improves tolerance of oilseed rape to cadmium stress. Front. Plant Sci. 11, 547133. doi: 10.3389/fpls.2020.547133 33193479 PMC7644955

[B8] Almuwayhi M. A. (2021). Effect of cadmium on the molecular and morpho-physiological traits of *Pisum sativum* L. Biotechnol. Biotechnol. Equip. 35, 1374–1384. doi: 10.1080/13102818.2021.1978318

[B9] Al-Qurainy F. Alameri A. A. Salim K. (2010). RAPD profile for the assessment of genotoxicity on a medicinal plant; *Eruca sativa* . J. Med. Plant Res. 4, 579–586. doi: 10.5897/JMPR10.062

[B10] Anarado C. E. Anarado C. J. O. Agwuna C. Okeke M. O. Okafor P. C. (2018). Phytoremediating potentials of *Sida acuta* and *Duranta erecta* for lead, cadmium, cobalt and zinc. Int. J. Sci. Res. 7, 969–971. doi: 10.21275/ART20192715

[B11] Asgari-Lajayer B. Ghorbanpour M. Nikabadi S. (2017). Heavy metals in contaminated environment: Destiny of secondary metabolite biosynthesis, oxidative status and phytoextraction in medicinal plants. Ecotoxicol. Environ. Saf. 145, 377–390. doi: 10.1016/j.ecoenv.2017.07.035 28759767

[B12] ATSDR (2013) Where is cadmium found? cadmium toxicity. Available at: https://www.atsdr.cdc.gov/csem/cadmium/Where-Cadmium-Found.html.

[B13] Azizollahi Z. Ghaderian S. M. Ghotbi-Ravandi A. A. (2019). Cadmium accumulation and its effects on physiological and biochemical characters of summer savory (*Satureja hortensis* L.). Int. J. Phytoremediation 21, 1241–1253. doi: 10.1080/15226514.2019.1619163 31140292

[B14] Bahl A. S. (2022) India’s alternative medicine industry grows, boosted by the covid-19 pandemic. times of India. Available at: https://timesofindia.indiatimes.com/blogs/voices/indias-alternative-medicine-industry-grows-boosted-by-the-covid-19-pandemic/ (Accessed November 25, 2022).

[B15] Banu Doğanlar Z. (2013). Metal accumulation and physiological responses induced by copper and cadmium in *Lemna gibba*, L. minor and *Spirodela polyrhiza* . Chem. Speciat. Bioavailab. 25, 79–88. doi: 10.3184/095422913X13706128469701

[B16] Bao A. Burritt D. J. Chen H. Zhou X. Cao D. Tran L.-S. P. (2019). The CRISPR/Cas9 system and its applications in crop genome editing. Crit. Rev. Biotechnol. 39, 321–336. doi: 10.1080/07388551.2018.1554621 30646772

[B17] Bauddh K. Singh R. P. (2012). Cadmium tolerance and its phytoremediation by two oil yielding plants *Ricinus communis* (L.) and *Brassica juncea* (L.) from the contaminated soil. Int. J. Phytoremediation 14, 772–785. doi: 10.1080/15226514.2011.619238 22908643

[B18] Benavides M. P. Gallego S. M. Tomaro M. L. (2005). Cadmium toxicity in plants. Braz. J. Plant Physiol. 17, 21–34. doi: 10.1590/S1677-04202005000100003

[B19] Bewley J. D. (1997). Seed germination and dormancy. Plant Cell 9, 1055–1066. doi: 10.1105/tpc.9.7.1055 12237375 PMC156979

[B20] Biswas T. Parveen O. Pandey V. P. Mathur A. Dwivedi U. N. (2020). Heavy metal accumulation efficiency, growth and centelloside production in the medicinal herb *Centella asiatica* (L.) urban under different soil concentrations of cadmium and lead. Ind. Crops Prod. 157, 112948. doi: 10.1016/j.indcrop.2020.112948

[B21] Cai Z. Xian P. Wang H. Lin R. Lian T. Cheng Y.. (2020). Transcription factor GmWRKY142 confers cadmium resistance by up-regulating the cadmium tolerance 1-like genes. Front. Plant Sci. 11, 724. doi: 10.3389/fpls.2020.00724 32582254 PMC7283499

[B22] Carvalho M. E. A. Gaziola S. A. Carvalho L. A. Azevedo R. A. (2021). Cadmium effects on plant reproductive organs: Physiological, productive, evolutionary and ecological aspects. Ann. Appl. Biol. 178, 227–243. doi: 10.1111/aab.12612

[B23] Chakrabarti M. Mukherjee A. (2021). Investigating the underlying mechanism of cadmium-induced plant adaptive response to genotoxic stress. Ecotoxicol. Environ. Saf. 209, 111817. doi: 10.1016/j.ecoenv.2020.111817 33383339

[B24] Chen Y. Zhi J. Zhang H. Li J. Zhao Q. Xu J. (2017). Transcriptome analysis of *Phytolacca americana* L. in response to cadmium stress. PLoS One 12, e0184681. doi: 10.1371/journal.pone.0184681 28898278 PMC5595333

[B25] Chen Q. Lu X. Guo X. Pan Y. Yu B. Tang Z.. (2018c). Differential responses to cd stress induced by exogenous application of Cu, zn or Ca in the medicinal plant *Catharanthus roseus* . Ecotoxicol. Environ. Saf. 157, 266–275. doi: 10.1016/j.ecoenv.2018.03.055 29626640

[B26] Chen K. Wang Y. Zhang R. Zhang H. Gao C. (2019). CRISPR/cas genome editing and precision plant breeding in agriculture. Annu. Rev. Plant Biol. 70, 667–697. doi: 10.1146/annurev-arplant-050718-100049 30835493

[B27] Chen L. Wan H. Qian J. Guo J. Sun C. Wen J.. (2018b). Genome-wide association study of cadmium accumulation at the seedling stage in rapeseed (*Brassica napus* L.). Front. Plant Sci. 9, 375. doi: 10.3389/fpls.2018.00375 29725340 PMC5917214

[B28] Chen K. Yu Y. Sun K. Xiong H. Yu C. Chen P.. (2018a). The miRNAome of ramie (*Boehmeria nivea* L.): identification, expression, and potential roles of novel microRNAs in regulation of cadmium stress response. BMC Plant Biol. 18, 369. doi: 10.1186/s12870-018-1561-5 30577815 PMC6303851

[B29] Citterio S. Santagostino A. Fumagalli P. Prato N. Ranalli P. Sgorbati S. (2003). Heavy metal tolerance and accumulation of cd, cr and Ni by *Cannabis sativa* l. Plant Soil 256, 243–252. doi: 10.1023/A:1026113905129

[B30] Cobbett C. Goldsbrough P. (2002). Phytochelatins and metallothioneins: roles in heavy metal detoxification and homeostasis. Annu. Rev. Plant Biol. 53, 159–182. doi: 10.1146/annurev.arplant.53.100301.135154 12221971

[B31] Dai H. Wei S. Pogrzeba M. Krzyżak J. Rusinowski S. Zhang Q. (2021). The cadmium accumulation differences of two *Bidens pilosa* l. ecotypes from clean farmlands and the changes of some physiology and biochemistry indices. Ecotoxicol. Environ. Saf. 209, 111847. doi: 10.1016/j.ecoenv.2020.111847 33388723

[B32] DalCorso G. Farinati S. Furini A. (2010). Regulatory networks of cadmium stress in plants. Plant Signal. Behav. 5, 663–667. doi: 10.4161/psb.5.6.11425 20404494 PMC3001555

[B33] DalCorso G. Fasani E. Manara A. Visioli G. Furini A. (2019). Heavy metal pollutions: State of the art and innovation in phytoremediation. Int. J. Mol. Sci. 20 (14), 3412. doi: 10.3390/ijms20143412 31336773 PMC6679171

[B34] Dang F. Lin J. Chen Y. Li G. X. Guan D. Zheng S. J.. (2019). A feedback loop between CaWRKY41 and H2O2 coordinates the response to *Ralstonia solanacearum* and excess cadmium in pepper. J. Exp. Bot. 70, 1581–1595. doi: 10.1093/jxb/erz006 30649526 PMC6416791

[B35] Deng X. Xia Y. Hu W. Zhang H. Shen Z. (2010). Cadmium-induced oxidative damage and protective effects of n-acetyl-L-cysteine against cadmium toxicity in *Solanum nigrum* l. J. Hazard. Mater. 180 (1-3), 722–729. doi: 10.1016/j.jhazmat.2010.04.099 20488618

[B36] Ding G.-H. Guo D.-D. Guan Y. Chi C.-Y. Liu B.-D. (2019). Changes of DNA methylation of *Isoetes sinensis* under Pb and Cd stress. Environ. Sci. pollut. Res. Int. 26, 3428–3435. doi: 10.1007/s11356-018-3864-3 30515690

[B37] Dobrikova A. G. Apostolova E. L. Hanć A. Yotsova E. Borisova P. Sperdouli I (2021). Cadmium toxicity in *Salvia sclarea* L.: An integrative response of element uptake, oxidative stress markers, leaf structure and photosynthesis. Ecotoxicol. Environ. Saf. 209, 111851. doi: 10.1016/j.ecoenv.2020.111851 33421673

[B38] Dresler S. Hanaka A. Bednarek W. Maksymiec W. (2014). Accumulation of low-molecular-weight organic acids in roots and leaf segments of *Zea mays* plants treated with cadmium and copper. Acta Physiol. Plant 36, 1565–1575. doi: 10.1007/s11738-014-1532-x

[B39] Dwivedi G. K. Upadhyay S. K. Mishra A. K. Singh A. K. (2013). Hyper accumulation of cadmium in *Phyllanthus amarus* L. a medicinal plant. Ind. J. Life Sci. 3, 21.

[B40] El-Dahiyat F. Rashrash M. Abuhamdah S. Abu Farha R. Babar Z.-U.-D. (2020). Herbal medicines: a cross-sectional study to evaluate the prevalence and predictors of use among Jordanian adults. J. Pharm. Policy Pract. 13, 2. doi: 10.1186/s40545-019-0200-3 31988754 PMC6971905

[B41] Faizan S. Haneef I. Kausar S. Perveen R. (2012). Germination and seedling growth of *Coriandrum sativum* L. under varying levels of mixed cadmium and copper. J. Funct. Environ. Bot. 2, 52–58. doi: 10.5958/j.2231-1742.2.1.008

[B42] Fan W. Liu C. Cao B. Qin M. Long D. Xiang Z.. (2018). Genome-wide identification and characterization of four gene families putatively involved in cadmium uptake, translocation and sequestration in mulberry. Front. Plant Sci. 9, 879. doi: 10.3389/fpls.2018.00879 30008726 PMC6034156

[B43] Fattahi B. Arzani K. Souri M. K. Barzegar M. (2019). Effects of cadmium and lead on seed germination, morphological traits, and essential oil composition of sweet basil (*Ocimum basilicum* L.). Ind. Crops Prod. 138, 111584. doi: 10.1016/j.indcrop.2019.111584

[B44] Feng S. Shen Y. Xu H. Dong J. Chen K. Xiang Y. (2021). RNA-Seq Identification of Cd Responsive Transporters Provides Insights into the Association of Oxidation Resistance and Cd Accumulation in *Cucumis sativus* L. Antioxidants (Basel) 10, 111584. doi: 10.3390/antiox10121973 PMC8750378 34943077

[B45] Fukuda N. Kitajima N. Terada Y. Abe T. Nakai I. Hokura A. (2020). Visible cellular distribution of cadmium and zinc in the hyperaccumulator *Arabidopsis halleri ssp. gemmifera* determined by 2-d X-ray fluorescence imaging using high-energy synchrotron radiation. Metallomics 12, 193–203. doi: 10.1039/c9mt00243j 31845691

[B46] Furze J. M. Rhodes M. J. Parr A. J. Robins R. J. Withehead I. M. Threlfall D. R. (1991). Abiotic factors elicit sesquiterpenoid phytoalexin production but not alkaloid production in transformed root cultures of *Datura stramonium* . Plant Cell Rep. 10, 111–114. doi: 10.1007/BF00232039 24221487

[B47] Fusco N. Micheletto L. Dal Corso G. Borgato L. Furini A. (2005). Identification of cadmium-regulated genes by cDNA-AFLP in the heavy metal accumulator *Brassica juncea* l. J. Exp. Bot. 56, 3017–3027. doi: 10.1093/jxb/eri299 16216843

[B48] Gall J. E. Boyd R. S. Rajakaruna N. (2015). Transfer of heavy metals through terrestrial food webs: a review. Environ. Monit. Assess. 187, 201. doi: 10.1007/s10661-015-4436-3 25800370

[B49] García de la Torre V. S. Coba de la Peña T. Lucas M. M. Pueyo J. J. (2022). Transgenic *Medicago truncatula* plants that accumulate proline display enhanced tolerance to cadmium stress. Front. Plant Sci. 13, 829069. doi: 10.3389/fpls.2022.829069 35154232 PMC8826176

[B50] Ghori N.-H. Ghori T. Hayat M. Q. Imadi S. R. Gul A. Altay V.. (2019). Heavy metal stress and responses in plants. Int. J. Environ. Sci. Technol. 16, 1807–1828. doi: 10.1007/s13762-019-02215-8

[B51] Ghosh R. Roy S. (2019). “Cadmium toxicity in plants: Unveiling the physicochemical and molecular aspects,” in Cadmium tolerance in plants. Eds. Hasanuzzaman M. Vara Prasad M. N. Nahar K. (London, UK: Academic Press), 223–246.

[B52] Gilbert S. F. (2000). Vegetative growth in developmental biology (Sunderland, Massachusetts, USA: Sinauer Associates.,Sunderland).

[B53] Gill S. S. Khan N. A. Tuteja N. (2012). Cadmium at high dose perturbs growth, photosynthesis and nitrogen metabolism while at low dose it up regulates sulfur assimilation and antioxidant machinery in garden cress (*Lepidium sativum* L.). Plant Sci. 182, 112–120. doi: 10.1016/j.plantsci.2011.04.018 22118622

[B54] Gómez Ariza J. L. García-Barrera T. García-Sevillano M. A. González-Fernández M. Gómez-Jacinto V. (2013). “Metallomics and metabolomics of plants under environmental stress caused by metals,” Heavy Metal Stress in Plants (eds). Gupta D. K. Corpas F. J. Palma J. M. (Berlin, Heidelberg: Springer Berlin Heidelberg), 173–201.

[B55] Greco M. Chiappetta A. Bruno L. Bitonti M. B. (2012). In *Posidonia oceanica* cadmium induces changes in DNA methylation and chromatin patterning. J. Exp. Bot. 63, 695–709. doi: 10.1093/jxb/err313 22058406 PMC3254685

[B56] Gupta P. Khatoon S. Tandon P. K. Rai V. (2014). Effect of cadmium on growth, bacoside a, and bacopaside I of *Bacopa monnieri* (L.), a memory enhancing herb. Sci. World J. 2014, 824586. doi: 10.1155/2014/824586 PMC3928858 24672380

[B57] Hangarter R. P. (2000) Vegetative growth. plants in motion. Available at: https://plantsinmotion.bio.indiana.edu/plantmotion/vegetative/veg.html.

[B58] Haque M. Biswas K. Sinha S. N. (2021). “Phytoremediation strategies of some plants under heavy metal stress,” in Plant stress physiology. Ed. Hossain A. (London, UK: IntechOpen).

[B59] Hasan M. K. Liu C. Wang F. Ahammed G. J. Zhou J. Xu M.-X.. (2016). Glutathione-mediated regulation of nitric oxide, s-nitrosothiol and redox homeostasis confers cadmium tolerance by inducing transcription factors and stress response genes in tomato. Chemosphere 161, 536–545. doi: 10.1016/j.chemosphere.2016.07.053 27472435

[B60] Hawrylak-Nowak B. Dresler S. Matraszek R. (2015). Exogenous malic and acetic acids reduce cadmium phytotoxicity and enhance cadmium accumulation in roots of sunflower plants. Plant Physiol. Biochem. 94, 225–234. doi: 10.1016/j.plaphy.2015.06.012 26115548

[B61] Huang X. Duan S. Wu Q. Yu M. Shabala S. (2020). Reducing cadmium accumulation in plants: Structure-function relations and tissue-specific operation of transporters in the spotlight. Plants 9, 223. doi: 10.3390/plants9020223 32050442 PMC7076666

[B62] Huang J. Wu X. Tian F. Chen Q. Luo P. Zhang F.. (2019). Changes in proteome and protein phosphorylation reveal the protective roles of exogenous nitrogen in alleviating cadmium toxicity in poplar plants. Int. J. Mol. Sci. 21, 278. doi: 10.3390/ijms21010278 31906144 PMC6982014

[B63] Huang S. Q. Xiang A. L. Che L. L. Chen S. Li H. Song J. B.. (2010). A set of miRNAs from *Brassica napus* in response to sulphate deficiency and cadmium stress. Plant Biotechnol. J. 8, 887–899. doi: 10.1111/j.1467-7652.2010.00517.x 20444207

[B64] Huang Y. Xi Y. Gan L. Johnson D. Wu Y. Ren D.. (2019). Effects of lead and cadmium on photosynthesis in *Amaranthus spinosus* and assessment of phytoremediation potential. Int. J. Phytoremediation 21, 1041–1049. doi: 10.1080/15226514.2019.1594686 31020865

[B65] Iqbal N. Nazar R. Umar S. (2016). “Evaluating the importance of proline in cadmium tolerance and its interaction with phytohormones,” in Osmolytes and plants acclimation to changing environment: Emerging omics technologies. Eds. Iqbal N. Nazar R. Khan N. A. (New Delhi, India: Springer India), 129–153.

[B66] Jamla M. Khare T. Joshi S. Patil S. Penna S. Kumar V. (2021). Omics approaches for understanding heavy metal responses and tolerance in plants. Curr. Plant Biol. 27, 100213. doi: 10.1016/j.cpb.2021.100213

[B67] Jian H. Yang B. Zhang A. Ma J. Ding Y. Chen Z.. (2018). Genome-wide identification of microRNAs in response to cadmium stress in oilseed rape (*Brassica napus* L.) using high-throughput sequencing. Int. J. Mol. Sci. 19, 1431. doi: 10.3390/ijms19051431 29748489 PMC5983666

[B68] Kapoor D. Rattan A. Bhardwaj R. Kaur S. (2016). Photosynthetic efficiency, ion analysis and carbohydrate metabolism in *Brassica juncea* plants under cadmium stress. J. Pharmacog. Phytochem. 5, 279–286.

[B69] Kenny C.-R. Ring G. Sheehan A. Mc Auliffe M. A.P. Lucey B. Furey A. (2022). Novel metallomic profiling and non-carcinogenic risk assessment of botanical ingredients for use in herbal, phytopharmaceutical and dietary products using HR-ICP-SFMS. Sci. Rep. 12, 17582. 36266322 10.1038/s41598-022-16873-1 PMC9584900

[B70] Khan N. You F. M. Datla R. Ravichandran S. Jia B. Cloutier S. (2020). Genome-wide identification of ATP binding cassette (ABC) transporter and heavy metal associated (HMA) gene families in flax (*Linum usitatissimum* L.). BMC Genomics 21, 722. doi: 10.1186/s12864-020-07121-9 33076828 PMC7574471

[B71] Khatamipour M. Piri E. Esmaeilian Y. Tavassoli A. (2011). Toxic effect of cadmium on germination, seedling growth and proline content of milk thistle (*Silybum marianum*). Ann. Biol. Res. 2, 527–532.

[B72] Khateeb W. (2013). Cadmium-induced changes in germination, seedlings growth, and DNA fingerprinting of *in vitro* grown *Cichorium pumilum* jacq. Int. J. Biol. 6, 65. doi: 10.5539/ijb.v6n1p65

[B73] Kilic S. (2017). Effects of cadmium-induced stress on essential oil production, morphology and physiology of lemon balm (*Melissa officinalis* l., lamiaceae). Appl. Ecol. Environ. Res. 15, 1653–1669. doi: 10.15666/aeer/1503_16531669

[B74] Komal T. Mustafa M. Ali Z. Kazi A. G. (2015). “Heavy metal uptake and transport in plants,” in Heavy metal contamination of soils: Monitoring and remediation. Eds. Sherameti I. Varma A. (Switzerland: Springer International Publishing), 181–194.

[B75] Kováčik J. Tomko J. Bačkor M. Repčák M.. (2006). *Matricaria chamomilla* is not a hyperaccumulator, but tolerant to cadmium stress. Plant Growth Reg. 50, 239–247. doi: 10.1007/s10725-006-9141-3

[B76] Krantev A. Yordanova R. Janda T. Szalai G. Popova L. (2008). Treatment with salicylic acid decreases the effect of cadmium on photosynthesis in maize plants. J. Plant Physiol. 165 (9), 920–931. doi: 10.1016/j.jplph.2006.11.014 17913285

[B77] Kubier A. Wilkin R. T. Pichler T. (2019). Cadmium in soils and groundwater: A review. Appl.Geochem. 108, 1–16. doi: 10.1016/j.apgeochem.2019.104388 32280158 PMC7147761

[B78] Kumar S. Trivedi P. K. (2018). Glutathione s-transferases: Role in combating abiotic stresses including arsenic detoxification in plants. Front. Plant Sci. 9, 751. doi: 10.3389/fpls.2018.00751 29930563 PMC5999759

[B79] Li D. He G. Tian W. Huang Y. Meng L. He Y.. (2021a) Genome-wide identification of metal tolerance genes in potato (Solanum tuberosum): response to two heavy metal stress. Available at: https://www.researchsquare.com/article/rs-166067/latest.pdf.

[B80] Li Z. Liu Z. Chen R. Li X. Tai P. Gong Z.. (2015). DNA Damage and genetic methylation changes caused by cd in *Arabidopsis thaliana* seedlings. Environ. Toxicol. Chem. 34, 2095–2103. doi: 10.1002/etc.3033 25914311

[B81] Li X. Mao X. Xu Y. Li Y. Zhao N. Yao J.. (2021b). Comparative transcriptomic analysis reveals the coordinated mechanisms of *Populus × canadensis* “Neva” leaves in response to cadmium stress. Ecotoxicol. Environ. Saf. 216, 112179. doi: 10.1016/j.ecoenv.2021.112179 33798869

[B82] Li F.-T. Qi J.-M. Zhang G.-Y. Lin L.-H. Fang P.-P. Tao A.-F.. (2013). Effect of cadmium stress on the growth, antioxidative enzymes and lipid peroxidation in two kenaf (*Hibiscus cannabinus* L.) plant seedlings. J. Integr. Agric. 12 (4), 610–620. doi: 10.1016/S2095-3119(13)60279-8

[B83] Li X. Zhao M. Guo L. Huang L. (2012). Effect of cadmium on photosynthetic pigments, lipid peroxidation, antioxidants, and artemisinin in hydroponically grown *Artemisia annua* . J. Environ. Sci. 24, 1511–1518. doi: 10.1016/S1001-0742(11)60920-0 23513695

[B84] Logani M. K. Davies R. E. (1980). Lipid oxidation: biologic effects and antioxidants–a review. Lipids 15, 485–495. doi: 10.1007/BF02534079 7401947

[B85] Lu Q. Chen S. Li Y. Zheng F. He B. Gu M. (2020). Exogenous abscisic acid (ABA) promotes cadmium (Cd) accumulation in *Sedum alfredii* hance by regulating the expression of Cd stress response genes. Environ. Sci. pollut. Res. Int. 27, 8719–8731. doi: 10.1007/s11356-019-07512-w 31912395

[B86] Luo J.-S. Gu T. Yang Y. Zhang Z. (2019). A non-secreted plant defensin AtPDF2.6 conferred cadmium tolerance *via* its chelation in *Arabidopsis* . Plant Mol. Biol. 100, 561–569. doi: 10.1007/s11103-019-00878-y 31053987

[B87] Luo H. Li H. Zhang X. Fu J. (2011). Antioxidant responses and gene expression in perennial ryegrass (*Lolium perenne* L.) under cadmium stress. Ecotoxicology 20, 770–778. doi: 10.1007/s10646-011-0628-y 21442247

[B88] Lux A. Martinka M. Vaculík M. White P. J. (2011b). Root responses to cadmium in the rhizosphere: a review. J. Exp. Bot. 62 (1), 21–37. doi: 10.1093/jxb/erq281 20855455

[B89] Lux A. Vaculík M. Martinka M. Lisková D. Kulkarni M. G. Stirk W. A.. (2011a). Cadmium induces hypodermal periderm formation in the roots of the monocotyledonous medicinal plant *Merwilla plumbea* . Ann. Bot. 107 (2), 285–292. doi: 10.1093/aob/mcq240 21118841 PMC3025738

[B90] Mahmud J. A. Hasanuzzaman M. Nahar K. Bhuyan M. H. M. B. Fujita M. (2018). Insights into citric acid-induced cadmium tolerance and phytoremediation in *Brassica juncea* l.: Coordinated functions of metal chelation, antioxidant defense and glyoxalase systems. Ecotoxicol. Environ. Saf. 147, 990–1001. doi: 10.1016/j.ecoenv.2017.09.045 29976011

[B91] Ma X. Su Z. Ma H. (2020). Molecular genetic analyses of abiotic stress responses during plant reproductive development. J. Exp. Bot. 71 (10), 2870–2885. doi: 10.1093/jxb/eraa089 32072177 PMC7260722

[B92] Mehes-Smith M. Nkongolo K. Cholewa E. (2013). “Coping mechanisms of plants to metal contaminated soil,” in Environmental change and sustainability. Eds. Silvern S. Young S. (London, UK: IntechOpen).

[B93] Mengdi X. Haibo D. Jiaxin L. Zhe X. Yi C. Xuan L.. (2020). Metabolomics reveals the “Invisible” detoxification mechanisms of *Amaranthus hypochondriacus* at three ages upon exposure to different levels of cadmium. Ecotoxicol. Environ. Saf. 195, 110520. doi: 10.1016/j.ecoenv.2020.110520 32213366

[B94] Miglani G. S. (2017). Genome editing in crop improvement: Present scenario and future prospects. J. Crop Improv. 31, 453–559. doi: 10.1080/15427528.2017.1333192

[B95] Mirosławski J. Paukszto A. (2018). Determination of the cadmium, chromium, nickel, and lead ions relays in selected polish medicinal plants and their infusion. Biol. Trace Elem. Res. 182, 147–151. doi: 10.1007/s12011-017-1072-5 28620726 PMC5808095

[B96] Mishra B. Sangwan R. S. Mishra S. Jadaun J. S. Sabir F. Sangwan N. S. (2014). Effect of cadmium stress on inductive enzymatic and nonenzymatic responses of ROS and sugar metabolism in multiple shoot cultures of ashwagandha (*Withania somnifera* dunal). Protoplasma 251, 1031–1045. doi: 10.1007/s00709-014-0613-4 24510215

[B97] Mishra S. Srivastava S. Tripathi R. D. Govindarajan R. Kuriakose S. V. Prasad M. N. V. (2006). Phytochelatin synthesis and response of antioxidants during cadmium stress in *Bacopa monnieri* L. Plant Physiol. Biochem. 44, 25–37. doi: 10.1016/j.plaphy.2006.01.007 16545573

[B98] Mobin M. Khan N. A. (2007). Photosynthetic activity, pigment composition and antioxidative response of two mustard (*Brassica juncea*) cultivars differing in photosynthetic capacity subjected to cadmium stress. J. Plant Physiol. 164, 601–610. doi: 10.1016/j.jplph.2006.03.003 16621132

[B99] Narender S. J. (2005) Heavy metals pollution of soil and plants due to sewage irrigation effect of heavy metals and sewage on seed germination and plant growth. (Doctoral dissertation). (Rohtak: Maharshi Dayanand University). Available at: https://shodhganga.inflibnet.ac.in/handle/10603/113472.

[B100] Nasim S. A. Dhir B. (2010). Heavy metals alter the potency of medicinal plants. Rev. Environ. Contam. Toxicol. 203, 139–149. doi: 10.1007/978-1-4419-1352-4_5 19957120

[B101] Navarro-León E. Oviedo-Silva J. Ruiz J. M. Blasco B. (2019). Possible role of HMA4a TILLING mutants of *Brassica rapa* in cadmium phytoremediation programs. Ecotoxicol. Environ. Saf. 180, 88–94. doi: 10.1016/j.ecoenv.2019.04.081 31078020

[B102] Niekerk L.-A. Carelse M. F. Bakare O. O. Mavumengwana V. Keyster M. Gokul A. (2021). The relationship between cadmium toxicity and the modulation of epigenetic traits in plants. Int. J. Mol. Sci. 22, 7046. doi: 10.3390/ijms22137046 34209014 PMC8268939

[B103] Nourbakhsh Rezaei S. R. Shabani L. Rostami M. Abdoli M. (2019). The effect of different concentrations of cadmium chloride on oxidative stress in shoot cultures of lemon balm. J. Plant Prod. 42, 509–522. doi: 10.22055/ppd.2019.24806.1567

[B104] Okem A. Southway C. Stirk W. A. Street R. A. Finnie J. F. Van Staden J. (2015). Effect of cadmium and aluminum on growth, metabolite content and biological activity in *Drimia elata* (Jacq.) hyacinthaceae. S. Afr. J. Bot. 98, 142–147. doi: 10.1016/j.sajb.2015.02.013

[B105] Oladeji O. (2016). The characteristics and roles of medicinal plants: Some important medicinal plants in Nigeria. Nat. Prod. Ind. J. 12, 102.

[B106] Paape T. Heiniger B. Santo Domingo M. Clear M. R. Lucas M. M. Pueyo J. J. (2021). Genome-wide association study reveals complex genetic architecture of cadmium and mercury accumulation and tolerance traits in *Medicago truncatula* . Front. Plant Sci. 12, 806949. doi: 10.3389/fpls.2021.806949 35154199 PMC8832151

[B107] Page V. Feller U. (2015). Heavy metals in crop plants: Transport and redistribution processes on the whole plant level. Agronomy 5, 447–463. doi: 10.3390/agronomy5030447

[B108] Pandey S. Gupta K. Mukherjee A. K. (2007). Impact of cadmium and lead on *Catharanthus roseus*–a phytoremediation study. J. Environ. Biol. 28, 655–662. 18380091

[B109] Pandita D. (2021). “CRISPR/Cas-mediated genome editing for improved stress tolerance in plants,” in Frontiers in plant-soil interaction. Eds. Aftab T. Hakeem K. R. (London, UK: Academic Press), 259–291.

[B110] Parmar P. Kumari N. Sharma V. (2013). Structural and functional alterations in photosynthetic apparatus of plants under cadmium stress. Bot. Stud. 54, 45. doi: 10.1186/1999-3110-54-45 28510881 PMC5430381

[B111] Patel A. H. (2006a) Heavy metal impact assessment study on growth and metabolism of medicinal plants. (Doctoral dissertation) (Ahmedabad: Gujarat University). Available at: https://shodhganga.inflibnet.ac.in/handle/10603/46164.

[B112] Patel J. G. (2006b) Study on relative toxicity of heavy metals to medicinal plants. (Doctoral dissertation). (Ahmedabad: Gujarat University). Available at: https://shodhganga.inflibnet.ac.in/handle/10603/45719.

[B113] Patel K. P. Patel K. M. (2012). Cadmium-induced changes in antioxidative enzyme activities and content of leaf pigments in *Cajanus cajan* (L.). Nat. Environ. pollut. Technol. 11, 47–50.

[B114] Patnaik A. Mohanty B. K. (2013). Toxic effect of mercury and cadmium on germination and seedling growth of *Cajanus cajan* l. (pigeon pea). Ann. Biol. Res. 4, 123–126.

[B115] Pena L. B. Pasquini L. A. Tomaro M. L. Gallego S. M. (2006). Proteolytic system in sunflower (*Helianthus annuus* L.) leaves under cadmium stress. Plant Sci. 171, 531–537. doi: 10.1016/j.plantsci.2006.06.003 25193651

[B116] Pereira M. P. Rodrigues L. C. de A. Corrêa F. F. de Castro E. M. Ribeiro V. E.. (2016). Cadmium tolerance in *Schinus molle* trees is modulated by enhanced leaf anatomy and photosynthesis. Trees 30, 807–814. doi: 10.1007/s00468-015-1322-0

[B117] Pongrac P. Serra T. S. Castillo-Michel H. Vogel-Mikuš K. Arčon I. Kelemen M. (2018). Cadmium associates with oxalate in calcium oxalate crystals and competes with calcium for translocation to stems in the cadmium bioindicator *Gomphrena claussenii* . Metallomics 10, 1576–1584. 30183791 10.1039/c8mt00149a

[B118] Rai V. Khatoon S. Bisht S. S. Mehrotra S. (2005). Effect of cadmium on growth, ultramorphology of leaf and secondary metabolites of *Phyllanthus amarus* schum. and thonn. Chemosphere 61, 1644–1650. doi: 10.1016/j.chemosphere.2005.04.052 15992855

[B119] Rai K. K. Pandey N. Meena R. P. Rai S. P. (2021). Biotechnological strategies for enhancing heavy metal tolerance in neglected and underutilized legume crops: A comprehensive review. Ecotoxicol. Environ. Saf. 208, 111750. doi: 10.1016/j.ecoenv.2020.111750 33396075

[B120] Rani S. Singh V. Sharma M. K. Sisodia R. (2021). GC–MS based metabolite profiling of medicinal plant-*Catharanthus roseus* under cadmium stress. Plant Physiol. Rep. 26, 491–502. doi: 10.1007/s40502-021-00595-z

[B121] Raza A. Habib M. Kakavand S. N. Zahid Z. Zahra N. Sharif R.. (2020). Phytoremediation of cadmium: Physiological, biochemical, and molecular mechanisms. Biology 9, 177. doi: 10.3390/biology9070177 32708065 PMC7407403

[B122] Rodrigues L. C. A. Martins J. P. R. de Almeida Júnior O. Guilherme L. R. G. Pasqual M. de Castro E. M. (2017). Tolerance and potential for bioaccumulation of *Alternanthera tenella* colla to cadmium under *in vitro* conditions. Plant Cell Tiss. Org. Cult. 130, 507–519. doi: 10.1007/s11240-017-1241-4

[B123] Rogowska A. Pączkowski C. Szakiel A. (2022). Modulation of steroid and triterpenoid metabolism in *Calendula officinalis* plants and hairy root cultures exposed to cadmium stress. Int. J. Mol. Sci. 23. doi: 10.3390/ijms23105640 PMC9145312 35628449

[B124] Romero-Puertas M. C. Palma J. M. Gómez M. Del Río L. A. Sandalio L. M. (2002). Cadmium causes the oxidative modification of proteins in pea plants. Plant Cell Environ. 25, 677–686. doi: 10.1046/j.1365-3040.2002.00850.x

[B125] Saggar S. Mir P. A. Kumar N. Chawla A. Uppal J. Shilpa S.. (2022). Traditional and herbal medicines: Opportunities and challenges. Pharmacognosy Res. 14, 107–114. doi: 10.5530/pres.14.2.15

[B126] Salarizadeh S. Kavousi H. R. Pourseyadi S. (2016). Effect of cadmium on germination characters and biochemical parameters of two Iranian ecotypes of cumin (*Cuminum cyminum* L.). J. Medicinal Plants By-Product 5, 15–22. doi: 10.22092/JMPB.2016.108919

[B127] Sanità di Toppi L. Gabbrielli R. (1999). Response to cadmium in higher plants. Environ. Exp. Bot. 41, 105–130. doi: 10.1016/S0098-8472(98)00058-6

[B128] Sarma H. Islam N. F. Prasad R. Prasad M. N. V. Ma L. Q. Rinklebe J. (2021). Enhancing phytoremediation of hazardous metal(loid)s using genome engineering CRISPR-Cas9 technology. J. Hazard. Mater. 414, 125493. doi: 10.1016/j.jhazmat.2021.125493 34030401

[B129] Sarry J.-E. Kuhn L. Ducruix C. Lafaye A. Junot C. Hugouvieux V.. (2006). The early responses of *Arabidopsis thaliana* cells to cadmium exposure explored by protein and metabolite profiling analyses. Proteomics 6, 2180–2198. doi: 10.1002/pmic.200500543 16502469

[B130] Sasaki A. Yamaji N. Yokosho K. Ma J. F. (2012). Nramp5 is a major transporter responsible for manganese and cadmium uptake in rice. Plant Cell 24, 2155–2167. doi: 10.1105/tpc.112.096925 22589467 PMC3442593

[B131] Seneviratne M. Rajakaruna N. Rizwan M. Madawala H. M. S. P. Ok Y. S. Vithanage M. (2019). Heavy metal-induced oxidative stress on seed germination and seedling development: a critical review. Environ. Geochem. Health 41, 1813–1831. doi: 10.1007/s10653-017-0005-8 28702790

[B132] Sethy S. K. Ghosh S. (2013). Effect of heavy metals on germination of seeds. J. Nat. Sci. Biol. Med. 4, 272–275. doi: 10.4103/0976-9668.116964 24082715 PMC3783763

[B133] Sharmila P. Kumari P. K. Singh K. Prasad N. V. S. R. K. Pardha-Saradhi P. (2017). Cadmium toxicity-induced proline accumulation is coupled to iron depletion. Protoplasma 254, 763–770. doi: 10.1007/s00709-016-0988-5 27311981

[B134] Shekari L. Aroiee H. Mirshekari A. Nemati H. (2019). Protective role of selenium on cucumber (*Cucumis sativus* L.) exposed to cadmium and lead stress during reproductive stage role of selenium on heavy metals stress. J. Plant Nutr. 42, 529–542. doi: 10.1080/01904167.2018.1554075

[B135] Shi G. Liu C. Cui M. Ma Y. Cai Q. (2012). Cadmium tolerance and bioaccumulation of 18 hemp accession. Appl. Biochem. Biotechnol. 168, 163–173. doi: 10.1007/s12010-011-9382-0 21938417

[B136] Singh S. Eapen S. D’Souza S. F. (2006). Cadmium accumulation and its influence on lipid peroxidation and antioxidative system in an aquatic plant, *Bacopa monnieri* L. Chemosphere 62, 233–246. doi: 10.1016/j.chemosphere.2005.05.017 15993469

[B137] Singh N. Mansoori A. Dey D. Kumar R. Kumar A. (2021). “Potential of metabolomics in plant abiotic stress management,” in Omics technologies for sustainable agriculture and global food security, vol. II . Eds. Kumar A. Kumar R. Shukla P. Patel H. K. (Singapore: Springer Singapore), 193–214.

[B138] Singh S. Parihar P. Singh R. Singh V. P. Prasad S. M. (2015). Heavy metal tolerance in plants: Role of transcriptomics, proteomics, metabolomics, and ionomics. Front. Plant Sci. 6, 1143. doi: 10.3389/fpls.2015.01143 26904030 PMC4744854

[B139] Singh V. Verma K. (2018). Metals from cell to environment: Connecting Metallomics with other omics. Open Journal of Plant Science 3, 001–014. doi: 10.1080/15226514.2016.1207598

[B140] Song Y. Jin L. Wang X. (2017). Cadmium absorption and transportation pathways in plants. Int. J. Phytoremediation 19, 133–141. doi: 10.1080/15226514.2016.1207598 27409403

[B141] Songmei L. Jie J. Yang L. Jun M. Shouling X. Yuanyuan T.. (2019). Characterization and evaluation of OsLCT1 and OsNramp5 mutants generated through CRISPR/cas9-mediated mutagenesis for breeding low cd rice. Rice Sci. 26, 88–97. doi: 10.1016/j.rsci.2019.01.002

[B142] So O. Oyewole S. O. Akinyemi O. Jimoh K. A. (2018). Medicinal plants and sustainable human health: a review. Hortic. Int. J. 2, 194–195. doi: 10.15406/hij.2018.02.00051

[B143] Souri M. K. Hatamian M. Tesfamariam T. (2019). Plant growth stage influences heavy metal accumulation in leafy vegetables of garden cress and sweet basil. Chem. Biol. Technol. Agric. 6, 1–7. doi: 10.1186/s40538-019-0170-3

[B144] Sridhar B. B. M. Diehl S. V. Han F. X. Monts D. L. Su Y. (2005). Anatomical changes due to uptake and accumulation of zn and cd in Indian mustard (*Brassica juncea*). Environ. Exp. Bot. 54, 131–141. doi: 10.1016/j.envexpbot.2004.06.011

[B145] Srivastava V. Sarkar A. Singh S. Singh P. de Araujo A. S. F. Singh R. P. (2017). Agroecological responses of heavy metal pollution with special emphasis on soil health and plant performances. Front. Environ. Sci. 5. doi: 10.3389/fenvs.2017.00064

[B146] Srivastava J. Yadav S. (2017). Cadmium phytoextraction and induced antioxidant gene response in *Moringa oleifera* lam. Am. J. Plant Physiol. 12, 58–70. doi: 10.3923/ajpp.2017.58.70

[B147] Sterckeman T. Thomine S. (2020). Mechanisms of cadmium accumulation in plants. Crit. Rev. Plant Sci. 39, 322–359. doi: 10.1080/07352689.2020.1792179

[B148] Street R. A. (2012). Heavy metals in medicinal plant products — an African perspective. S. Afr. J. Bot. 82, 67–74. doi: 10.1016/j.sajb.2012.07.013

[B149] Tang L. Mao B. Li Y. Lv Q. Zhang L. Chen C.. (2017). Knockout of OsNramp5 using the CRISPR/Cas9 system produces low cd-accumulating indica rice without compromising yield. Sci. Rep. 7, 14438. doi: 10.1038/s41598-017-14832-9 29089547 PMC5663754

[B150] Tchounwou P. B. Yedjou C. G. Patlolla A. K. Sutton D. J. (2012). Heavy metal toxicity and the environment. Exp. 101, 133–164. doi: 10.1007/978-3-7643-8340-4_6 PMC4144270 22945569

[B151] Thakur S. Choudhary S. Majeed A. Singh A. Bhardwaj P. (2020). Insights into the molecular mechanism of arsenic phytoremediation. J. Plant Growth Regul. 39, 532–543. doi: 10.1007/s00344-019-10019-w

[B152] Thomine S. Wang R. Ward J. M. Crawford N. M. Schroeder J. I. (2000). Cadmium and iron transport by members of a plant metal transporter family in *Arabidopsis* with homology to nramp genes. Proc. Natl. Acad. Sci. U.S.A 97, 4991–4996. doi: 10.1073/pnas.97.9.4991 10781110 PMC18345

[B153] Thongchai A. Meeinkuirt W. Taeprayoon P. Chelong I.-A. (2021). Effects of soil amendments on leaf anatomical characteristics of marigolds cultivated in cadmium-spiked soils. Sci. Rep. 11 (1), 15909. doi: 10.1038/s41598-021-95467-9 34354195 PMC8342601

[B154] Trivedi N. G. (2003) A study on responses of adhatoda vasica l to heavy metal. (Doctoral dissertation) (Ahmedabad: Gujarat University). Available at: https://shodhganga.inflibnet.ac.in/handle/10603/46182.

[B155] Venegas-Rioseco J. Ginocchio R. Ortiz-Calderón C. (2021). Increase in phytoextraction potential by genome editing and transformation: A review. Plants 11. doi: 10.3390/plants11010086 PMC8747683 35009088

[B156] Verdoy D. Coba de la Peña T. Redondo F. J. Lucas M. M. Pueyo J. J. (2006). Transgenic *Medicago truncatula* plants that accumulate proline display nitrogen-fixing activity with enhanced tolerance to osmotic stress. Plant Cell Environ. 29, 1913–1923. doi: 10.1111/j.1365-3040.2006.01567.x 16930317

[B157] Wagner G. J. (1993). Accumulation of cadmium in crop plants and its consequences to human health. Adv. Agron. 51, 173–212. doi: 10.1016/S0065-2113(08)60593-3

[B158] Wang T. Li Y. Fu Y. Xie H. Song S. Qiu M.. (2019). Mutation at different sites of metal transporter gene OsNramp5 affects Cd accumulation and related agronomic traits in rice (*Oryza sativa* L.). Front. Plant Sci. 10, 1081. doi: 10.3389/fpls.2019.01081 31572408 PMC6749076

[B159] Wang C. Qiao F. Wang M. Wang Y. Xu Y. Qi X. (2023). PvERF104 confers cadmium tolerance in Arabidopsis: Evidence for metal-responsive element-binding transcription factors. Environ. Exp. Bot. 206, 105167. doi: 10.1016/j.envexpbot.2022.105167

[B160] Weerakoon S. R. (2019). “Genetic engineering for metal and metalloid detoxification,” in Transgenic plant technology for remediation of toxic metals and metalloids. Ed. Prasad M. N. V. (London, UK: Academic Press), 23–41.

[B161] Wójcik M. Vangronsveld J. D’Haen J. Tukiendorf A. (2005). Cadmium tolerance in *Thlaspi caerulescens*: II. localization of cadmium in *Thlaspi caerulescens* . Environ. Exp. Bot. 53, 163–171. doi: 10.1016/S0098-8472(04)00047-4

[B162] Xu J. Yin H. Li X. (2009). Protective effects of proline against cadmium toxicity in micropropagated hyperaccumulator, *Solanum nigrum* L. Plant Cell Rep. 28, 325–333. doi: 10.1007/s00299-008-0643-5 19043719

[B163] Yadav S. K. (2010). Heavy metals toxicity in plants: An overview on the role of glutathione and phytochelatins in heavy metal stress tolerance of plants. S. Afr. J. Bot. 76, 167–179. doi: 10.1016/j.sajb.2009.10.007

[B164] Yang J.-L. Liu L.-W. Gong Y.-Q. Huang D.-Q. Wang F. He L.-L. (2007). Analysis of genomic DNA methylation level in radish under cadmium stress by methylation-sensitive amplified polymorphism technique. Zhi Wu Sheng Li Yu Fen Zi Sheng Wu Xue Xue Bao 33, 219–226. 17556809

[B165] Yang L. Yao J. Sun J. Shi L. Chen Y. Sun J. (2020). The Ca2+ signaling, glu, and GABA responds to cd stress in duckweed. Aquat. Toxicol. 218, 105352. doi: 10.1016/j.aquatox.2019.105352 31790938

[B166] Ye Z. H. Baker A. J. M. Wong M. H. Willis A. J. (1997). Zinc, lead and cadmium tolerance, uptake and accumulation by *Typha latifolia* . New Phytol. 136 (3), 469–480. doi: 10.1046/j.1469-8137.1997.00759.x 33863011

[B167] Ye P. Wang M. Zhang T. Liu X. Jiang H. Sun Y.. (2020). Enhanced cadmium accumulation and tolerance in transgenic hairy roots of *Solanum nigrum* L. expressing iron-regulated transporter gene IRT1. Life 10. doi: 10.3390/life10120324 PMC7761695 33287205

[B168] Youssef N. A. (2021). Changes in the morphological traits and the essential oil content of sweet basil (*Ocimum basilicum* L.) as induced by cadmium and lead treatments. Int. J. Phytoremediation 23 (3), 291–299. doi: 10.1080/15226514.2020.1812508 32997524

[B169] Yuan J. Bai Y. Chao Y. Sun X. He C. Liang X.. (2018). Genome-wide analysis reveals four key transcription factors associated with cadmium stress in creeping bentgrass (*Agrostis stolonifera* L.). Peer J. 6, e5191. doi: 10.7717/peerj.5191 30083437 PMC6071620

[B170] Yuan J. Fu H. Wang X. (2021). Coupled metabolome with physiology unveiled mechanisms for cadmium affecting active ingredients synthesis in *Salvia miltiorrhiza*, a non-Cd-hyperaccumulator. Res. Square 1, 1–22. doi: 10.21203/rs.3.rs-886213/v1

[B171] Yu X. Liang L. Xie Y. Tang Y. Tan H. Zhang J.. (2022). Comparative analysis of Italian lettuce (*Lactuca sativa* L. var. ramose) transcriptome profiles reveals the molecular mechanism on exogenous melatonin preventing cadmium toxicity. Genes 13. doi: 10.3390/genes13060955 PMC9223142 35741717

[B172] Yu G. Ma J. Jiang P. Li J. Gao J. Qiao S.. (2019). “The mechanism of plant resistance to heavy metal,” in IOP Conference Series: Earth and Environmental Science IOP science, Vol. 310. 052004. doi: 10.1088/1755-1315/310/5/052004

[B173] Yu R. Tang Y. Liu C. Du X. Miao C. Shi G. (2017). Comparative transcriptomic analysis reveals the roles of ROS scavenging genes in response to cadmium in two pak choi cultivars. Sci. Rep. 7, 9217. doi: 10.1038/s41598-017-09838-2 28835647 PMC5569009

[B174] Zaheer I. E. Ali S. Rizwan M. Farid M. Shakoor M. B. Gill R. A.. (2015). Citric acid assisted phytoremediation of copper by *Brassica napus* L. Ecotoxicol. Environ. Saf. 120, 310–317. doi: 10.1016/j.ecoenv.2015.06.020 26099461

[B175] Zahra W. Rai S. N. Birla H. Singh S. S. Rathore A. S. Dilnashin H.. (2020). “Economic importance of medicinal plants in Asian countries,” in Bioeconomy for sustainable development. Ed. Keswani C. (Singapore: Springer), 359–377.

[B176] Zaid A. Mohammad F. Fariduddin Q. (2020). Plant growth regulators improve growth, photosynthesis, mineral nutrient and antioxidant system under cadmium stress in menthol mint (*Mentha arvensis* L.). Physiol. Mol. Biol. Plants 26 (1), 25–39. doi: 10.1007/s12298-019-00715-y 32158118 PMC7036404

[B177] Zayneb C. Bassem K. Zeineb K. Grubb C. D. Noureddine D. Hafedh M.. (2015). Physiological responses of fenugreek seedlings and plants treated with cadmium. Environ. Sci. pollut. Res. Int. 22 (14), 10679–10689. doi: 10.1007/s11356-015-4270-8 25752634

[B178] Zhang P. Wang R. Ju Q. Li W. Tran L.-S. P. Xu J. (2019). The R2R3-MYB transcription factor MYB49 regulates cadmium accumulation. Plant Physiol. 180, 529–542. doi: 10.1104/pp.18.01380 30782964 PMC6501104

[B179] Zhang F.-Q. Wang Y.-S. Lou Z.-P. Dong J.-D. (2007). Effect of heavy metal stress on antioxidative enzymes and lipid peroxidation in leaves and roots of two mangrove plant seedlings (*Kandelia candel* and *Bruguiera gymnorrhiza*). Chemosphere 67 (1), 44–50. doi: 10.1016/j.chemosphere.2006.10.007 17123580

[B180] Zhang Y. Xu S. Yang S. Chen Y. (2015). Salicylic acid alleviates cadmium-induced inhibition of growth and photosynthesis through upregulating antioxidant defense system in two melon cultivars (*Cucumis melo* L.). Protoplasma 252 (3), 911–924. doi: 10.1007/s00709-014-0732-y 25398649

[B181] Zhao S. Duo L. (2015). Bioaccumulation of cadmium, copper, zinc, and nickel by weed species from municipal solid waste compost. Pol. J. Environ. Stud. 24, 413–417. doi: 10.15244/pjoes/28960

[B182] Zhao Q. Wang H. Du Y. Rogers H. J. Wu Z. Jia S.. (2020). MSH2 and MSH6 in mismatch repair system account for soybean (*Glycine max* (L.) merr.) tolerance to cadmium toxicity by determining DNA damage response. J. Agric. Food Chem. 68, 1974–1985. doi: 10.1021/acs.jafc.9b06599 31971785

[B183] Zhou X.-M. Zhao P. Wang W. Zou J. Cheng T.-H. Peng X.-B.. (2015). A comprehensive, genome-wide analysis of autophagy-related genes identified in tobacco suggests a central role of autophagy in plant response to various environmental cues. DNA Res.: Int. J. Rapid Publ. Rep. Genes Genomes 22 (4), 245–257. doi: 10.1093/dnares/dsv012 PMC4535619 26205094

[B184] Zou C. Lu T. Wang R. Xu P. Jing Y. Wang R.. (2022). Comparative physiological and metabolomic analyses reveal that Fe_3_O_4_ and ZnO nanoparticles alleviate cd toxicity in tobacco. J. Nanobiotechnology 20, 302. doi: 10.1186/s12951-022-01509-3 35761340 PMC9235244

